# Experience-dependent cortical plasticity in response to formal schooling: Effects on networks for reading and mathematics

**DOI:** 10.1016/j.dcn.2026.101749

**Published:** 2026-05-25

**Authors:** Floor Vandecruys, Maaike Vandermosten, Bert De Smedt

**Affiliations:** a Parenting and Special Education Research Unit, KU Leuven, Belgium; b Experimental ORL, Department of Neurosciences, KU Leuven, Belgium; c Leuven Brain Institute, KU Leuven, Belgium

**Keywords:** Cortical structure, MRI, Neuroplasticity, Early childhood, School cut-off design, Reading, Mathematics

## Abstract

Between ages 5–10, children undergo substantial structural brain development. These changes coincide with the transition from preschool to primary school. The extent to which this school transition drives the observed structural brain changes remains unclear. We used a school cut-off design to disentangle the effects of formal schooling and age-related maturation on children’s brain development by comparing children who attended formal education in primary school and children of similar age but who remained in preschool. Sixty-four children (36 schooling, *Med*_age_ = 68.5 months, 19 girls; 28 non-schooling, *Med*_age_ = 66 months, 19 girls) were assessed at two timepoints one year apart. Mixed-effects models were applied to MRI data to examine longitudinal changes in cortical volume, thickness, and surface area in regions implicated in reading and mathematics. Significant schooling effects were observed in bilateral – but predominantly left – temporal, parietal and inferior frontal regions associated with emergent reading and mathematics. More specifically, reading gains were associated with increased volume and thickness in right inferior frontal gyrus, with increased thickness in left superior parietal and increased volume in right inferior temporal cortices. Mathematical gains were correlated with increased thickness in left inferior parietal cortex. Children who exhibited larger increases in volume and thickness also showed stronger improvement in their academic achievement. Our findings demonstrate that the transition to formal education is associated with bilateral changes in cortical gray matter, highlighting the role of formal schooling – beyond maturation – in shaping the developing brain and offering an intriguing example of experience-dependent neuroplasticity.

## 1 Introduction

Between ages 5 and 10, children undergo remarkable changes in neurocognitive functioning and brain maturation (Galván, 2010). This period is marked by increased total brain volume (Bethlehem et al., 2022, Lebel and Beaulieu, 2011, Sowell, 2004) and regionally heterogenous patterns of cortical gray matter development, with regions showing increases, decreases, or relative stability (Ducharme et al., 2016, Frangou et al., 2022, Gennatas et al., 2017, Mills et al., 2016, Phan et al., 2021, Schilling et al., 2023, Sowell, 2004). These structural brain changes coincide with critical milestones in early childhood, including the transition from preschool to formal education in primary school, where children explicitly learn fundamental skills such as reading and mathematics. Unlike spoken language, which develops naturally, reading and mathematics require explicit instruction. Indeed, our brains are not biologically predisposed for these skills; rather, they are culturally-transmitted skills that reshape the brain as they are acquired (Dehaene and Cohen, 2007). This process, known as experience-dependent neuroplasticity, highlights how brain regions adapt their function as children learn new skills (Johnson and de Haan, 2011). In recent years, a growing body of research increasingly acknowledges the role of environmental factors in shaping brain structure (Economou et al., 2024, Jednoróg et al., 2012, Noble et al., 2015, Romeo et al., 2018). However, one key environmental influence remains largely underexplored in this research line: the experience of formal schooling on structural brain development. While longitudinal brain imaging studies have followed children as they transition to primary school, a major limitation of these studies is that they cannot disentangle effects of age-related maturational development from those of schooling. As a result, we do not know which changes in brain structure during this developmental period are attributable to age-related maturation, to schooling, or to both. To address this, the current brain imaging study applies, for the first time, the school cut-off design to examine the impact of schooling on children’s structural brain development.

While a considerable amount of research has focused on investigating gray matter correlates of school-taught abilities, such as reading and mathematics, relatively little is known about the specific impact of formal schooling on the cortical structures supporting these abilities. Studies in children (ages 5–14) have identified associations between reading abilities and gray matter volume (GMV) in regions within the typical reading network (Chyl, Fraga-González, et al., 2021, for a review), such as bilateral inferior frontal gyri (Chyl et al., 2019, Richlan et al., 2013), left superior temporal gyrus (Wang et al., 2020), and ventral occipito-temporal regions, including the (left) fusiform gyrus (Simon et al., 2013). However, some studies have reported divergent findings, either failing to observe these associations or identifying additional regions (Chyl et al., 2019, Jednoróg et al., 2015, Torre and Eden, 2019). Reading ability has also been linked to cortical thickness in the left supramarginal gyrus (Xia et al., 2018) and bilateral temporal cortices (Perdue et al., 2020, Xia et al., 2018) as well as to surface area in bilateral fusiform gyri (Beelen et al., 2019). Longitudinal investigations tracking children from preschool to primary school have demonstrated that GMV increases predominantly in left-hemispheric temporal and temporoparietal regions (Phan et al., 2021, ages 6–8). Additionally, increased cortical thickness in the left inferior frontal cortex has been associated with phonological skill development (Lu et al., 2006, ages 6–8), a key predictor of successful reading acquisition (Melby-Lervåg et al., 2012, Wagner and Torgesen, 1987).

Studies, albeit in older children (ages 9–15), have also linked mathematical performance to gray matter structures. Specifically, cross-sectional associations have been observed with GMV of the right fusiform gyrus (Polspoel et al., 2020), the left inferior parietal cortex, including the intraparietal sulcus (IPS) (Li et al., 2013), left middle and superior temporal gyri (Suárez-Pellicioni et al., 2021), bilateral hippocampus (Wilkey et al., 2018) and right inferior frontal gyrus (Wilkey et al., 2018). Longitudinal data have indicated that GMV in regions including the left IPS (Price et al., 2016, ages 7–8), bilateral ventral occipito-temporal cortex, posterior parietal cortices (Evans et al., 2015, ages 8–14), and right hippocampus (Supekar et al., 2013, ages 8–9) predict improvements in mathematical skills. However, Suárez-Pellicioni et al. (2021), ages 10–15) reported that higher GMV in the left middle/superior temporal gyri predicted smaller subtraction gains and did not predict longitudinal gains in multiplication skill.

To the best of our knowledge, there is only one gray matter study in the field of mathematical cognition that longitudinally examined children in the transition from preschool to primary school (Kuhl et al., 2020). This study found that children who showed greater improvements in mathematical performance also exhibited reduced cortical thinning in the right temporal pole and left middle occipital gyrus (Kuhl et al., 2020). Given that both cortical regions typically exhibit age-related cortical thinning (Ducharme et al., 2016), the authors proposed that pattern may reflect a deceleration of cortical thinning in children who perform better, potentially as a result of increased engagement of training.

A major limitation to the abovementioned longitudinal studies is that they cannot isolate schooling-induced effects from concurrent maturational influences. Addressing this gap requires experimental designs that leverage environmental differences between groups (e.g., intervention studies), which provide the strongest evidence for causal relationships between environmental influences and brain development (Steele and Zatorre, 2018). There is a small number of studies that has investigated the effect of (educational) interventions on gray matter structures, and these have focused exclusively on reading (Perdue et al., 2022). Intensive reading interventions in children with dyslexia (ages 7 – 11) have demonstrated gray matter volume increases in the left fusiform gyrus, left precuneus, right hippocampus and right cerebellum (Krafnick et al., 2011). Similarly, Romeo et al. (2018) found that responders to a reading intervention exhibited greater cortical thickening than non-responders in bilateral middle/inferior temporal cortices, supramarginal/angular gyri, precentral regions and the right superior temporal gyrus. However, Partanen et al. (2021) found no evidence of gray matter plasticity after a reading intervention. More recently, Economou et al. (2024) reported significant cortical thickening in the left supramarginal gyrus among children at risk for dyslexia following a tablet-based phonics intervention. Additionally, they found significant cortical thinning for the control groups in the right inferior and left superior temporal gyri, while no thinning was observed for the intervention group. To the best of our knowledge, only one study has investigated the effect of a mathematical intervention in conjunction with structural MRI collection (Supekar et al., 2013). However, structural scans were acquired only prior to the intervention and were used to assess whether baseline brain structure could predict subsequent performance gains. As a result, the study did not examine neuroplasticity in response to the intervention.

In sum, existing cross-sectional and longitudinal studies have examined the neural correlates of reading and mathematics and their development over time, but these studies cannot disentangle effects of age-related maturational development from those of schooling. Research on experience-dependent plasticity suggests that specific cortical structures are malleable in response to intensive training, as demonstrated by intervention studies. However, such interventions are typically conducted in structured settings with children experiencing learning difficulties, making their findings difficult to generalize to typically developing children. As a result, we do not know how the brain structures involved in reading and mathematics are shaped by formal schooling – a critical experience in early childhood.

To address this gap in our understanding of how schooling shapes cortical structures, the school cut-off design has been put forward as a clever quasi-experimental approach (Morrison et al., 2019). It has been used to examine the impact of schooling on brain activity (e.g., Brod et al., 2017) and we will use it for the first time to study cortical structures underlying reading and mathematics. This design leverages the arbitrary cut-off that schools use for school entry, that is the child’s birth date. Children who are born right before or right after this cut-off date (January 1st in our country), are similar in age, but are enrolled in different school years. This design enables us to isolate the unique effects of schooling and maturation on children's development (Morrison et al., 2019) and serves as a natural experiment providing robust evidence for causal inferences (Miller et al., 2016). Using such a school cut-off design, Brod et al. (2017) examined response inhibition during a go/no-go task and found that the schooling group exhibited larger activation increases in the right superior posterior parietal cortex. Using the same task, McKay et al. (2022) observed that the schooling group demonstrated a larger difference in bilateral frontal cortex activation between correct and incorrect responses, indicating enhanced response monitoring. In contrast, Nolden et al. (2021) investigated schooling effects on memory performance but found no significant activation changes from pretest to posttest. Finally, Davidson et al. (2023) investigated schooling effects on working memory and reported increased brain activation in the left inferior frontal gyrus (IFG) and the left inferior parietal lobule (IPL) for the schooling group.

The present employs a school cut-off design to disentangle the effects of schooling from those of age-related maturation on gray matter structures in networks of reading and mathematics in a sample of five-year-old children. To this end, we compared two groups of similarly aged children: a "relatively young" first-grade group that received formal instruction in primary school (born between October and December 2015) and a "relatively old" preschool group that remained in a play-oriented preschool setting (born between January and March 2016). In the educational context of Flanders (Belgium), nearly all children (98%) attend preschool (ages 2.5–6 years), which is fully subsidized by the government. This preschool environment provides only a non-formal introduction to reading and counting (e.g., distinguishing letters from numbers), in accordance with Flemish government guidelines (http://www.ond.vlaanderen.be). This is different from first grade, where children are explicitly taught to read and write simple words and sentences. For mathematics, the key focus in first grade is on understanding numbers up to 20 as well as addition and subtraction. Importantly, in Flanders (Belgium) there is no systematic holding back of children before they start school.

We investigated whether gray matter development in several predefined regions was driven by schooling effects or by age-related maturation. We selected a set of regions-of-interest (ROIs) by focusing on those regions that have been structurally or functionally related to early reading and mathematics. We focused on three metrics of cortical structures. We analyzed overall (sub-)cortical volume and its two subcomponents: cortical thickness and cortical surface area. Cortical thickness and surface area follow distinct developmental trajectories, with surface area strongly shaped prenatally (Jha et al., 2019, Kapellou et al., 2006) and cortical thickness showing dynamic changes as a function of age and in line with cognitive development (Frangou et al., 2022, Sowell, 2004). While cortical thinning typically begins around age three (Bethlehem et al., 2022, Walhovd et al., 2016), skill acquisition can lead to localized thickening, although the exact underlying mechanisms remain debated (Ducharme et al., 2016, Lövdén et al., 2013, Tamnes et al., 2017). Based on this evidence, we hypothesized that experience-dependent plasticity (i.e., schooling effects) would be observed primarily in cortical thickness and cortical volume rather than in surface area. The limited evidence about region-specific gray matter development during this age range does not allow us to make hypotheses about the direction of these effects. We also examined whether observed schooling effects on gray matter structure were associated with behavioral gains in reading and mathematics.

## 2 Methods

### 2.1 Participants

The present study was carried out in the context of a larger longitudinal study, in which also diffusion-weighted MRI data (Vandecruys et al., 2024), functional MRI data (Vandecruys et al., *in review*) and more extensive behavioral data were collected, none of which were analyzed and reported here. This research was approved by the Medical Ethical Committee of the University of Leuven (S64318). Written informed consent was obtained from the parents of all participants, and verbal assent was provided by the children, in accordance with the Declaration of Helsinki. The current study involved two timepoints of data collection (pretest and posttest). At the first measurement point (T1) or pretest, conducted during summer 2021, all children attended preschool. One year later, at the second measurement point (T2) or posttest, half of the sample attended the first grade of primary school, whereas the other half remained in preschool.

We recruited, at T1, 64 children from preschool, allocated to a non-schooling (*n* = 28; *Med*_age_ = 66 months; range = 64–67 months, 19 girls) and schooling (*n* = 36; *Med*_age_ = 68.5 months; range = 65–70 months, 19 girls) group, respectively. The age groups under study were specifically selected because their date of birth fell on either side of the official school entry cut-off (January 1) in Flanders (Belgium). As a result, and following the logic of the school cut-off design (Morrison et al., 2019), we created two groups that were similar in age, but different in the amount of formal instruction they received. In particular, children in first grade have received massive structured and formal reading and arithmetic instruction. Children in preschool, on the other hand, received a very limited informal introduction in learning to count and read. We selected typically developing, monolingual children, without any medical history or history of developmental problems. Children who had a heart pacemaker or any other metallic foreign body were excluded due to standard MRI safety contraindications. The groups were matched on sex and parental educational attainment, the latter being recognized as a strong predictor of children’s cognitive and academic outcomes (Davis-Kean et al., 2021).

### 2.2 Materials and procedure

#### 2.2.1 Behavioral assessment

***Early reading.*** Children’s early reading abilities were examined using a word reading task, that is frequently used, standardized reading assessment in Flanders: the “Drie-Minuten-Test” (DMT). Children were asked to read as many words as possible, correctly, within 1 min. Originally, the DMT consists of three word lists, each read within one minute. In the present study, we only administered one word list was administered (for one minute). Consequently, normative scores were not calculated, and analyses were based on raw scores. Words were presented in a list and they were all monosyllabic. The final score on this reading assessment was the total number of correctly read words within one minute. The task was discontinued when a child was unable to read any of the first three words within the first fifteen seconds.

***Early mathematics.*** Children’s early mathematics skills were assessed using an arithmetic task addressing basic addition and subtraction problems. The task was divided into four blocks of increasing difficulty. The first block consisted of four basic addition and subtraction problems, which were presented by using stones. The stones were put in a box, after which a second number of stones was added or subtracted. The experimenter told the children using number words how many stones there were in the box and how many stones there were added or subtracted. Children were asked how many stones were left in the box. The problems of the second, third, and fourth block were not presented with stones but as symbolic representations. The second block involved four problems below 10 (e.g., 6–4). The third block comprised five problems that required the child to count above 10 (e.g., 9 + 3). Finally, the fourth block included five problems between 10 and 30 (e.g., 19–5). The final score was the number of correctly solved problems. The task was discontinued if a child was unable to answer any item correctly within one block.

***Verbal ability.*** To measure our participants’ receptive and expressive verbal ability, we administered the “Word Classes” subtest of the Clinical Evaluation of Language Fundamentals Preschool 2 (Dutch version) (CELF-preschool-2-NL; Semel et al., 2012). Children were asked to reason about which two out of three or four visually presented items belong together. This subtest evaluates the ability to understand relationships between words based on semantic class features and is therefore a representative measure of verbal ability. Nineteen items were presented to the child, and two points could be earned for each item. The first point was awarded upon the child’s successful identification of the two images that belonged together. The second point was awarded if the child also provided a correct rationale for why these images belonged together. The total raw score, with a maximum of 38, was included in our analyses.

***Spatial Ability.*** We administered the Matrix Reasoning subtest of the Dutch Wechsler Intelligence Scale for Children, third edition (Wechsler, 2011) to characterize our participants’ spatial ability. Children were asked to indicate which of four or five visually presented items would fit into the empty spot of an incomplete colored matrix or visual pattern. The test consisted of a maximum of 29 items and the total raw score was included in our analyses.

#### 2.2.2 MRI data acquisition

All images were acquired on a 3 T MRI scanner (Philips, Eindhoven, The Netherlands) with a SENSE 32-channel head-coil, located at the Department of Radiology of the University Hospital in Leuven, Belgium. Anatomical 3D T1-weighted images were acquired using a CS-SENSE TFE (compressed sensing-sensitivity encoding turbo field echo) sequence with the following parameters: 240 sagittal slices, 0.9 mm^3^ isotropic voxel size, repetition time/echo time (ms) = 9.1/4.2, flip angle 90°, 284 × 270 acquisition matrix, acquisition time = 3 min 30 s. Prior to the actual MRI session, children completed a practice session in which they got used to the scanner environment and protocol (Theys et al., 2014). Children’s heads were stabilized by using washcloths to minimize head motion.

Prior to data preprocessing, two independent researchers – blinded to participants' group membership – conducted a visual quality assessment of all T1-weighted images using the Blumenthal’s scale (Blumenthal et al., 2002). While the initial sample consisted of 64 participants assessed at two time points, not all children completed both MRI sessions. One child did not complete the first timepoint scan, and eight children did not complete the second timepoint scan due to scheduling difficulties or reluctance to undergo scanning. As a result, 119 T1-weighted datasets were available for quality assessment. Scans were excluded if rated as severe by at least one researcher, based on the presence of pronounced artifacts such as ringing and blurring. In total, 18 out of 119 datasets (13 at pretest and 5 at posttest) were excluded (15%), leaving 101 high-quality scans for further analysis. Interrater reliability, calculated using Cohen’s weighted kappa, was 0.76, indicating substantial agreement. Note that not all participants had complete pre-post usable datasets (complete data were available for *n* = 24 in the schooling group and *n* = 18 in the non-schooling group). Nevertheless, all high-quality scans were retained in the analyses, given that our choice of statistical framework (linear mixed effects models) is compatible with missing observations (Bernal-Rusiel et al., 2013).

#### 2.2.3 MRI data (pre-)processing

Pre-processing of the raw T1 images was performed via parallel processing in GNU parallel (Tange, 2018) using the recon-all function of the FreeSurfer 6.0 image analysis suite (Fischl, 2012), which is documented and freely available online at https://surfer.nmr.mgh.harvard.edu/. This processing stream includes motion correction (Reuter et al., 2010), removal of non-brain tissue using a hybrid watershed/surface deformation procedure (Ségonne et al., 2004), automated Talairach transformation, nonparametric nonuniform intensity normalization (Sled et al., 1998), tessellation of the gray/white matter boundary, automated topology correction (Fischl et al., 2001, Segonne et al., 2007) and surface deformation following intensity gradients to optimally place the gray/white and gray/cerebrospinal fluid borders at the location where the greatest shift in intensity defines the transition to the other tissue class (Dale et al., 1999, Dale and Sereno, 1993, Fischl and Dale, 2000). Each cortical model was registered to a spherical atlas using individual cortical folding patterns to match cortical geometry across participants (Dale et al., 1999). Full technical details of these procedures are described in prior publications (Dale et al., 1999, Fischl et al., 1999, Fischl et al., 2002).

The T1 images were then processed using the longitudinal analysis stream of FreeSurfer 6.0, which has demonstrated greater sensitivity than the cross-sectional pipeline in detecting cortical changes in longitudinal designs (Reuter et al., 2012) and has been frequently used in developmental studies (Economou et al., 2024, Linkersdörfer et al., 2015, Romeo et al., 2018, Schel and Klingberg, 2017, Wierenga et al., 2014). This process included multiple steps. First, an unbiased within-subject template space and image were created using robust, inverse consistent registration (Reuter et al., 2010). Furthermore, processing steps such as skull stripping, Talairach transforms, atlas registration as well as spherical surface maps and parcellations were then initialized with common information from the within-subject template, significantly increasing reliability and statistical power (Reuter et al., 2012). Data from participants with only one available timepoint were also processed using the longitudinal stream to ensure that all images included in statistical analysis undergo the same processing steps (Bernal-Rusiel et al., 2013).

Three important measures were extracted for further analyses. First, measures of gray matter volume were extracted from the subcortical segmentations. Second, from the surface-based cortical parcellations, measurements for cortical thickness (CT) and surface area (SA) were extracted. CT was quantified as the distance between the white surface (white and gray matter boundary) and the pial surface (gray matter and outer cerebrospinal fluid/dura boundary) of the cortex, while SA was quantified as the size of the white surface (Fischl and Dale, 2000, Fischl et al., 1999). Measures of CT and SA derived from the FreeSurfer surface-based processing stream have been validated and shown good test–retest reliability across multiple sites, scanner manufacturers and field strengths (Haddad et al., 2023, Han et al., 2006, Reuter et al., 2012). Regional cortical thickness and surface area measurements were obtained from ROIs selected as part of FreeSurfer’s automatic parcellation using the Desikan-Killiany atlas (Desikan et al., 2006). This atlas remains the most widely used in developmental studies of cortical morphometry (Bethlehem et al., 2022, Collins et al., 2022, Tamnes et al., 2017), and therefore provides the most appropriate framework for ensuring replicability and facilitating comparisons with previous research.

**Fig. 1:**
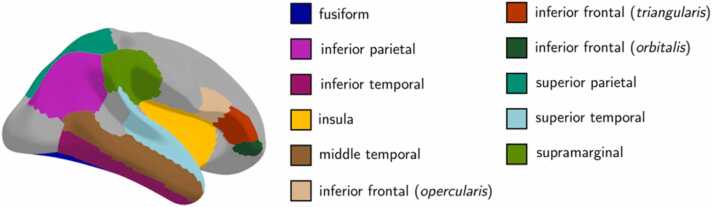
3D cortical surface rendering of ROIs considered in the current study. The color palette corresponds to the default FreeSurfer color scheme, with the exception of the fusiform gyrus, which was adjusted to improve visual clarity. Only the right hemisphere is shown. Hippocampus is not included in this figure.

Given the aim of this study to examine changes in brain structure in response to formal schooling versus age-related maturation, ROIs were predefined based on their functional relevance in emerging reading and mathematics. More specifically, we focused on a set of brain regions that are part of the reading and mathematical brain networks. In line with findings from independent research groups, this led to the selection of: temporal cortex (inferior, middle and superior temporal gyri), occipito-temporal cortex (fusiform gyrus), parietal cortex (supramarginal gyrus, angular gyrus, inferior and superior parietal gyrus), inferior frontal gyrus (*pars opercularis*, *pars triangularis*, *pars orbitalis*), insula and hippocampus (Evans et al., 2015, Jednoróg et al., 2015, Karipidis et al., 2021, Linkersdörfer et al., 2015, Perdue et al., 2020, Phan et al., 2021, Polspoel et al., 2020, Price et al., 2016, Suárez-Pellicioni et al., 2021, Xia et al., 2018, Yamada et al., 2011). Given the evidence of bilateral engagement in early reading and mathematics, both from structural (Li et al., 2013, Vandermosten et al., 2015, Walton et al., 2018) and functional (Davis et al., 2009, Raschle et al., 2012, Yamada et al., 2011) neuroimaging studies, as well as the observation that a left-hemispheric lateralization appears only at a later age (Qiu et al., 2011, Rivera et al., 2005, Yamada et al., 2011), both left- and right-hemispheric regions were investigated. Fig. 1 shows a 3D cortical rendering of the included ROIs in the current study, based on the Desikan-Killiany atlas (Desikan et al., 2006), and visualized with the *ggseg3d* package in R (Mowinckel and Vidal-Piñeiro, 2020).

### 2.3 Statistical analyses

All statistical analyses were conducted in R (version 4.2.1) (R Core Team, 2022). Both frequentist and Bayesian statistics are reported. The analyses followed a three-step approach. Firstly, group comparisons (non-schooling versus schooling) were performed to examine demographic characteristics. Mann-Whitney *U* tests were used for continuous data that did not follow a normal distribution, Pearson Chi-Squared tests were applied to categorical data, and independent samples *t*-tests were conducted for continuous data with a normal distribution. Effect sizes for parametric comparisons are reported as Cohen’s *d* values with values of 0.20, 0.40 and 0.80 representing a small, medium and large effect, respectively (Cohen, 2013). For non-parametric Mann-Whitney *U* tests, the rank-biserial correlation coefficient was reported, with values deviating further from zero indicating stronger effects (Willson, 1976). Additionally, Bayesian equivalents of these tests were calculated to quantify the evidence supporting the absence of a group difference (i.e., group matching, *BF*_01_).

Secondly, linear mixed-effects models were built for each predefined ROI and for each structural measure using the *lme4* package in R (Bates et al., 2015) to investigate the effects of age-related maturation and schooling. Timepoint (pretest versus posttest) and group (non-schooling versus schooling) were included as fixed categorical within- and between- subjects factors, respectively. A random intercept for participants was also incorporated into the model. For all linear mixed-effect models, Type III-analysis of variance tables were computed to obtain *F*-statistics and *p*-values using the Satterthwaite’s degrees of freedom approximation (Kuznetsova et al., 2017). To accommodate for issues with model diagnostics, such as non-normal or nonsymmetric residual distribution, robust linear mixed-effect models were applied (Koller, 2016). In all models, statistical inference was conducted by calculating 95% confidence intervals and *p*-values using parametric bootstrapping (1000 iterations) as implemented in the *parameters* R package (Lüdecke et al., 2020). Both main effects of timepoint as well as group-by-timepoint interaction effects are reported. In models where cortical volume was the dependent variable, total intracranial volume was included as control variable. Similarly, if regional surface area was the dependent variable, total hemispheric surface area was included as control variable. Age was not included as a covariate in the primary analyses. In a school cut-off design, group membership is determined by birth date. Consequently, the schooling group was by design systematically slightly older (approximately 3 months) than the non-schooling group. This age difference is therefore inherent to the quasi-experimental design rather than an incidental group imbalance. Statistically adjusting for age in this context would remove variance intrinsically linked to the schooling factor and may lead to overfitting or unstable parameter estimates due to the collinearity between age and group. Importantly, the age range of the sample was narrow (maximum six months), further limiting independent age-related variance.

Thirdly, for ROIs that demonstrated a schooling effect in the MRI analyses (i.e., a significant group-by-timepoint interaction effect), follow-up pairwise comparisons of the estimated marginals means (*emmeans* package, Lenth, 2017) were conducted with false discovery rate (FDR) correction (q = 0.05) (Benjamini and Hochberg, 1995). Standardized regression coefficients (*β*) were reported as effect sizes for all models.

For those ROIs that demonstrated a schooling-related interaction effect, we subsequently computed change scores (posttest – pretest) for the specific cortical parameter(s) (i.e., volume, thickness, or surface area) that showed the interaction. These longitudinal changes in cortical structure were then correlated with corresponding longitudinal changes in behavioral performance. Correlations were also corrected using the false discovery rate (FDR) (q = 0.05).

## 3 Results

### 3.1 Participant and group characteristics

Participant and group characteristics are summarized in Table 1. The two groups did not differ significantly with respect to sex, socioeconomic status, head motion, total intracranial volume and total hemispheric surface area. Although the group difference in age was minimal (2–3 months), it was significant, yet unavoidable given our cut-off design. Behavioral measures from pretest and posttest are shown in Table 2 and Table 3, respectively. Behavioral test results were well-distributed with no floor or ceiling effects. Large standard deviations highlight the large individual differences that exist at preschool. All measures had good to high reliability.

**Table 1:** Demographic participant and group characteristics. Significant group differences are presented in bold.

**Variables**	**Group**				**Test statistic (df)**	***p***	***BF***_**01**_^f^
	**Non-schooling**		**Schooling**				
	***n***	**Summary statistic**^a^	***n***	**Summary statistic**^a^			
**Sex** (female/male)	28	19/9	36	19/17	*χ*^*2*^ (1) = 1.485	.223^e^	1.599
**Socioeconomic status**^b^ Very low/low/middle/high	28	0/2/11/15	36	1/8/15/12	*χ*^*2*^ (3) = 4.621	.202^e^	1.382
**Head motion at pretest**^g^ None/mild/moderate/severe	28	1/14/9/4	35	3/11/12/9	*χ*^*2*^ (3) = 2.971	.396^e^	3.725
**Head motion at posttest**^g^ None/mild/moderate/severe	21	1/10/8/2	35	3/18/11/3	*χ*^*2*^ (3) = 0.490	.921^e^	8.993
**Left total surface area at pretest** (mm^3^)	24	80 (56 – 101)	26	82 (71 – 95)	*t*(48) = -0.953	.346^d^	2.438
**Right total surface area at pretest** (mm^3^)	24	80 (58 – 100)	26	82 (70 – 94)	*t*(48) = -0.816	.419^d^	2.691
**Left total surface area at posttest** (mm^3^)	19	83 (73 – 101)	32	84 (71 – 97)	*t*(49) = -0.558	.579^d^	3.057
**Right total surface area at posttest** (mm^3^)	19	83 (73 – 101)	32	84 (71 – 99)	*t*(49) = -0.536	.594^d^	3.086
**ICV at pretest**^h^ (mm^3^)	24	1367 (1203 – 1659)	26	1382 (1156 – 1605)	*t*(48) = -0.427	.671^d^	3.280
**ICV at posttest** (mm^3^)	19	1381 (1203 – 1659)	32	1396 (1156 – 1605)	*t*(49) = -0.450	.655^d^	3.195
**Age at pretest** (months)	28	66 (64 – 67)	36	68.50 (65 – 70)	*W* = 68.50	**< .001**^c^	0.001
**Age at posttest** (months)	23	78 (76 – 79)	35	81 (77 – 82)	*W* = 46.50	**< .001**^c^	0.002

a Median/Mean (range) or occurrence.

b Assessment was based on parental education level (very low = no extra degree after primary school, low = no extra degree after secondary school, middle = professional bachelor degree, high = master degree or PhD).

c Independent samples Mann-Whitney *U* test.

d Independent samples *t*-test.

e Chi-squared test.

f Bayes’ factors in support of **no** group difference are presented (null hypothesis, BF_01_). **BF**_**01**_**between 0–3**: anecdotal support; **between 3–10**: moderate support; **between 10–30**: strong support; **between 30–100**: very strong support; **> 100**: extremely strong support for no group differences (see Andraszewicz et al., 2015, for the classification).

g Datasets with severe motion judgment were excluded from further analyses (18 out of 119 datasets in total).

h Intracranial volume was calculated using an atlas-based estimation approach implemented in FreeSurfer.

**Table 2:** Descriptive statistics from pretest. Significant group differences are presented in bold.

**PRETEST**	**Group**											***α***^b^	**Statistic(df)**	***p***	**Effect size**	***BF***_**10**_^c^
	**Non-schooling**						**Schooling**									
	***n***	***M***	**Med**	**SD**	**Range**	***n***		***M***	**Med**	**SD**	**Range**					
Arithmetic^a^	28	2.86	2.00	2.29	1–10	36		5.17	4.00	3.982	0 – 17	.872	*W* = 305.00	**.007**	*r* = -0.395	2.25
Word reading^a^	28	2.36	0	11.52	0–61	36		1.08	0	2.750	0–11	/	*W* = 460.00	0.352	*r* = -0.087	0.34
Verbal ability^a^	27	28.59	28.00	4.46	18–36	35		29.74	31.00	4.231	19–38	.717	*t*(60) = -1.037	0.304	*d* = -0.266	0.41
Spatial ability^a^	27	14.89	15.00	3.37	9–22	35		16.34	16.00	4.69	8–26	.804	*t*(60) = -1.362	0.178	*d* = -0.349	0.57

Note.

a Variable has no normal distribution.

b Cronbach’s alpha.

c Bayes’ factors in support of a group difference are presented.

**Table 3:** Descriptive statistics from posttest. Significant group differences are presented in bold.

**POSTTEST**	**Group**										***α***^b^	**Statistic(df)**	***p***	**Effect size**	***BF***_**10**_^c^
	**Non-schooling**					**Schooling**									
	***n***	***M***	**Med**	**SD**	**Range**	***n***	***M***	**Med**	**SD**	**Range**					
Arithmetic^a^	23	7.35	7.00	4.12	1–16	35	14.09	14.00	3.68	6–18	.915	*W* = 95.00	**< .001**	*r* = -0.764	243.71
Word reading^a^	23	5.26	1.00	15.97	0–77	35	42.51	43.00	17.96	11–82	/	*W* = 35.50	**< .001**	*r* = -0.912	352.41
Verbal ability^a^	23	31.87	32.00	4.46	14–36	35	32.31	33.00	3.10	25–38	.715	*W* = 391.50	0.866	*r* = -0.027	0.30
Spatial ability^a^	23	20.09	20.00	4.46	9–29	35	20.20	21.00	4.76	10–27	.849	*t*(56) = -0.091	0.928	*d* = -0.024	0.27

Note.

a Variable has no normal distribution.

b Cronbach’s alpha.

c Bayes’ factors in support of a group difference are presented.

### 3.2 Linear mixed-effects models

Longitudinal changes in cortical volume, cortical thickness and surface area from pre- to posttest were compared in the schooling and non-schooling group by means of mixed-effects models to disentangle effects of schooling from effects of age-related maturation.

In Supplementary Figure 1, we showed that cortical volume was most closely related to cortical surface area (correlations ranging from *r* = 0.75, left IFG_orb_ to *r* = 0.97, left supramarginal), which was in line with previous research (Panizzon et al., 2009, Winkler et al., 2010). Correlations between cortical volume and cortical thickness ranged from *r* = 0.15 (right inferior parietal) to *r* = 0.66 (right superior temporal). Lastly, correlations between surface area and thickness ranged from *r* = -0.22 (right insula) to *r* = 0.31 (right superior temporal).

#### 3.2.1 Volume

**Fig. 2:**
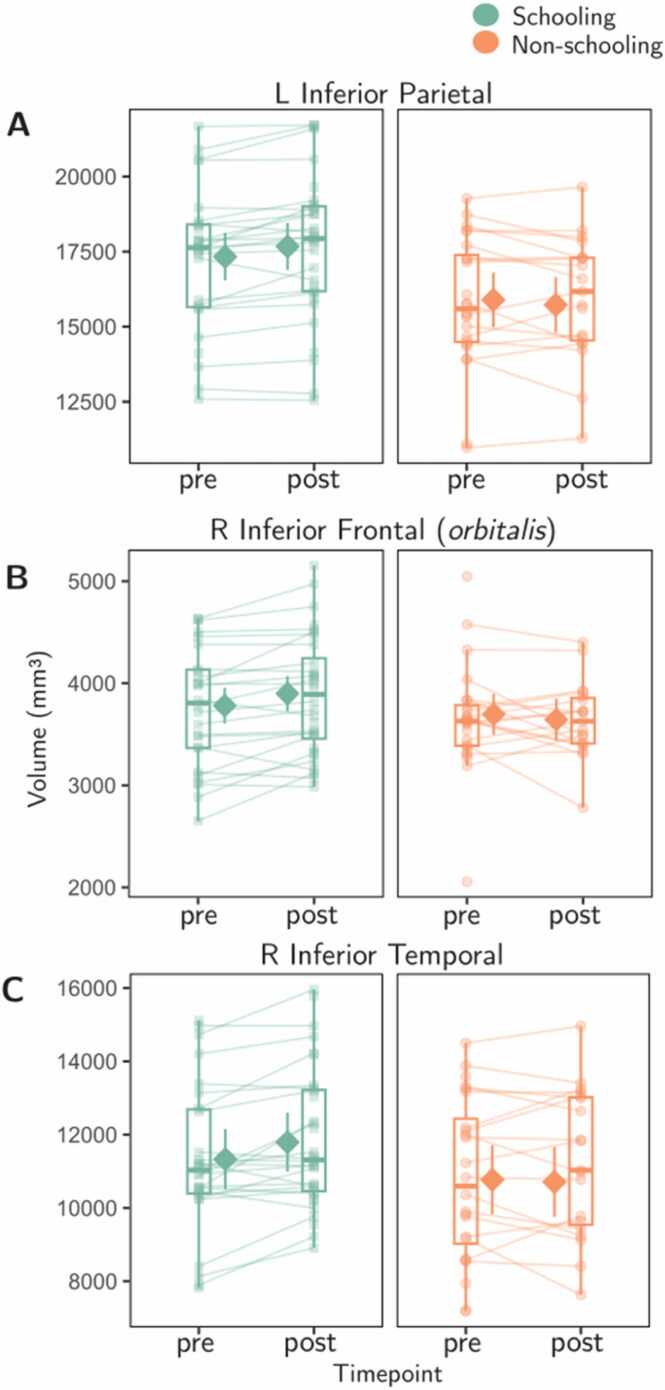
Significant group-by-timepoint interaction effects on volume of the A) left inferior parietal cortex, B) right inferior frontal gyrus (pars orbitalis), and C) right inferior temporal gyrus. Boxplots and semitransparent dots and lines represent individual subject longitudinal changes. The solid diamonds represent model-predicted volume values with 95% confidence bands across the two time points for the two groups.

As shown in Table 4, group-by-timepoint interaction effects were found on three ROIs, namely the **left** inferior parietal cortex (**IPC**), **right** inferior frontal gyrus (*pars orbitalis*, **IFG**_**orb**_) and **right** inferior temporal gyrus (**ITG**). Follow-up analyses indicated that for these ROIs, significant increased volume was found over time for the schooling group, and not for the non-schooling group. Post-hoc pairwise comparisons of the estimated marginal means are presented in Table 5. All effects remained significant after applying FDR-correction. Fig. 2 shows the significant group-by-timepoint interaction effects.

**Table 4:** Statistical results of significant group-by-timepoint interaction effects for parameter volume. Standardized regression coefficients (β) are given, with 95% confidence intervals.

**ROI**	**Std. Coeff. (*****β)*****[95% CI]**	**Statistic(df)**	***p***
**Left IPC**	0.10 [0.02, 0.17]	*t*(96) = 2.632	**.008**
**Right IFG**_**orb**_	0.15 [0.02, 0.28]	*t*(96) = 2.262	**.024**
**Right ITG**	0.12 [0.01, 0.23]	*t*(96) = 2.118	**.034**

**Table 5:** Post-hoc pairwise comparisons of the estimated marginal means of the significant group-by-timepoint interaction effects for parameter volume.

**ROI**	**Group**	**Estimate [95% asymp. CI]**	***z*****-ratio**	***p*****(*****p***_**fdr**_**)**
**Left IPC**	Schooling	ΔVOL = 342 mm^3^ [94, 590]	2.71	**.007 (.014)**
	Non-schooling	ΔVOL = -166 mm^3^ [-453, 120]	-1.14	.255 (.255)
**Right IFG**_**orb**_	Schooling	ΔVOL = 118 mm^3^ [21, 215]	2.39	**.017 (.033)**
	Non-schooling	ΔVOL = -52 mm^3^ [-164, 59]	-0.92	.358 (.358)
**Right ITG**	Schooling	ΔVOL = 463 mm^3^ [145, 780]	2.86	**.004 (.008)**
	Non-schooling	ΔVOL = -61 mm^3^ [-428, 305]	-0.33	.742 (.742)

Note. ΔVOL = difference (post – pre) in volume in mm^3^.

#### 3.2.2 Cortical thickness

**Fig. 3:**
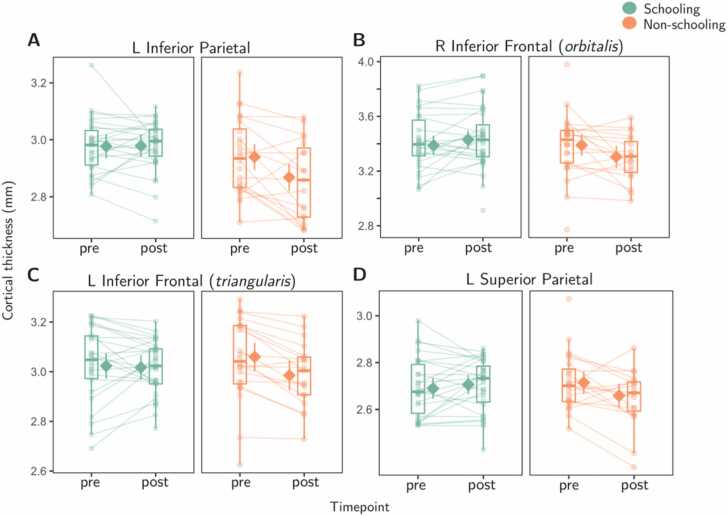
Significant group-by-timepoint interaction effects on cortical thickness for A) left inferior parietal cortex, B) right inferior frontal gyrus (pars orbitalis), C) left inferior frontal gyrus (pars triangularis), and D) left superior parietal cortex. Boxplots and semitransparent dots and lines represent individual subject longitudinal changes. The solid diamonds represent model-predicted thickness values with 95% confidence bands across the two time points for the two groups.

We found group-by-timepoint interaction effects on five ROIs, namely the **left IPC, right IFG**_**orb**_, **left** IFG **(***pars triangularis*, **IFG**_**tri**_**)**, **left SPC** and **right ITG** (Table 6). Follow-up analyses showed in these five regions significant cortical thinning over time for the non-schooling group, while no cortical thinning was observed in the schooling group. Post-hoc pairwise comparisons of the estimated marginal means are presented in Table 7. After applying the FDR-correction, the effect in the non-schooling group was no longer significant for the right ITG (*p*_fdr_ =.092). Fig. 3 visualizes the significant group-by-timepoint interaction effects.

**Table 6:** Statistical results of significant group-by-timepoint interaction effects for parameter cortical thickness. Standardized regression coefficients (β) are given, with 95% confidence intervals.

**ROI**	**Std. Coeff. (*****β)*****[95% CI]**	**Statistic(df)**	***p***
**Left IPC**	0.29 [0.08, 0.49]	*t*(97) = 2.789	**.005**
**Right IFG**_**orb**_	0.28 [0.07, 0.48]	*t*(97) = 2.700	**.007**
**Left IFG**_**tri**_	0.23 [0.05, 0.46]	*t*(97) = 2.477	**.013**
**Left SPC**	0.28 [0.04, 0.52]	*t*(97) = 2.312	**.021**
**Right ITG**	0.25 [0.01, 0.49]	*t*(97) = 2.099	**.036**

**Table 7:** Post-hoc pairwise comparisons of the estimated marginal means of the significant group-by-timepoint interaction effects for parameter cortical thickness.

**ROI**	**Group**	**Estimate [95% asymp. CI]**	***z*****-ratio**	***p*****(*****p***_**fdr**_**)**
**Left IPC**	Schooling	ΔCT = 1 *μ*m [-31, 35]	0.10	.917 (.917)
	Non-schooling	ΔCT = -71 *μ*m [-110, −32]	-3.60	**< .001 (<.001)**
**Right IFG**_**orb**_	Schooling	ΔCT = 4 *μ*m [-2, 10]	1.35	.177 (.177)
	Non-schooling	ΔCT = -9 *μ*m [-16, −2]	-2.40	**.016 (.032)**
**Left IFG**_**tri**_	Schooling	ΔCT = -5 *μ*m [-41, 30]	-0.32	.749 (.749)
	Non-schooling	ΔCT = -75 *μ*m [-116, −33]	-3.55	**< .001 (<.001)**
**Left SPC**	Schooling	ΔCT = 16 *μ*m [-23, 56]	0.80	.424 (.424)
	Non-schooling	ΔCT = -55 *μ*m [-101, −9]	-2.36	**.018 (.036)**
**Right ITG**	Schooling	ΔCT = 23 *μ*m [-27, 73]	0.91	.365 (.365)
	Non-schooling	ΔCT = -59 *μ*m [-117, −0.9]	-1.99	**.046** (.092)

Note. ΔCT = difference (post – pre) in cortical thickness in μm.

#### 3.2.3 Cortical surface area

**Fig. 4:**
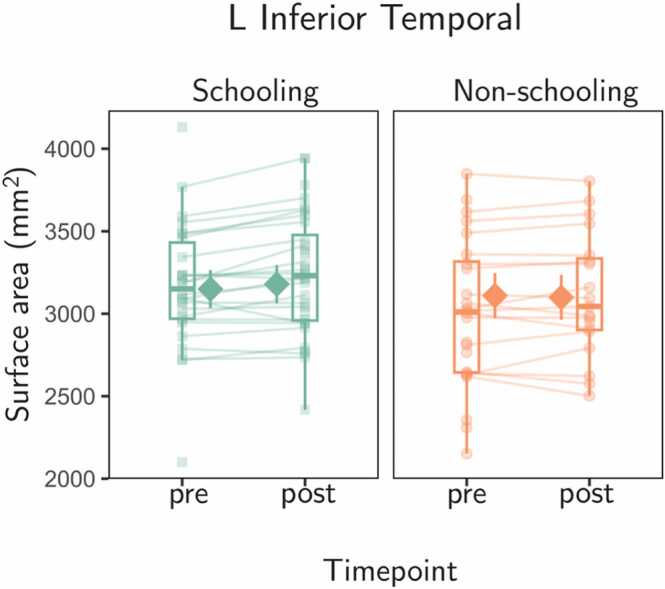
Significant group-by-timepoint interaction effect on surface area of the left inferior temporal gyrus. Boxplots and semitransparent dots and lines represent individual subject longitudinal changes. The solid diamonds represent model-predicted area values with 95% confidence bands across the two time points for the two groups.

One significant group-by-timepoint interaction effect was found, namely for the **left ITG** (*β* = 0.05, 95% CI [0.01, 0.09], *t*(96) = 2.15, *p* = .031), showing that the schooling group exhibited an increase in surface area over time (ΔSA = 30 mm^2^, 95% asymptotic CI [3.9, 55.4], *z* = 2.26, *p* = .024, *p*_fdr_ =.048), while the non-schooling group did not (ΔSA = −11 mm^2^, 95% asymptotic CI [-39.4, 17.4], *z* = -0.76, *p* = .449, *p*_fdr_ =.449). This effect remained significant after FDR-correction. Fig. 4 visualizes this group-by-timepoint effect.

### 3.3 Longitudinal brain-behavior correlations in the schooling group

For ROIs that demonstrated a schooling effect (i.e., left IPC volume/thickness, right IFG_orb_ volume/thickness, right ITG volume, left SPC thickness, left IFG_tri_ thickness, left ITG surface area), we calculated in the schooling group Pearson correlations between the longitudinal change of the significant cortical parameters and the longitudinal change in reading/mathematics. Correlations were corrected using the FDR (q = 0.05).

**Fig. 5:**
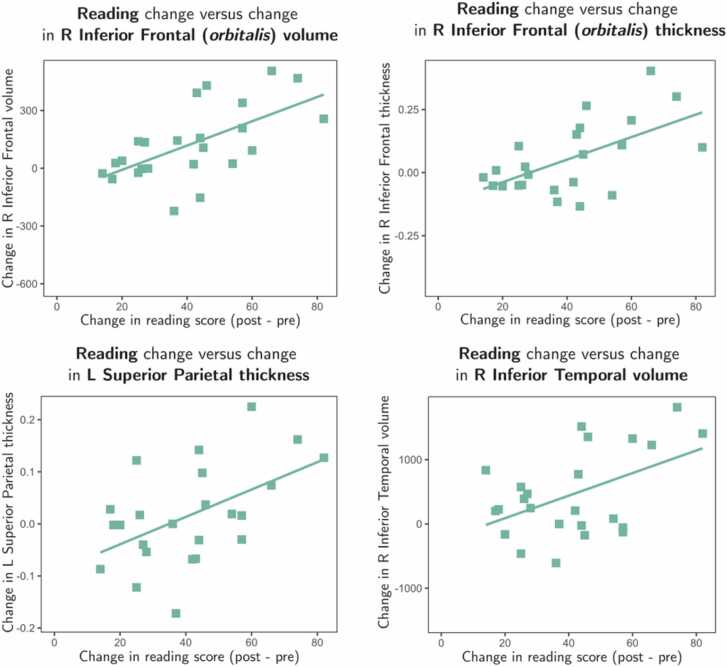
Grouped scatterplots of significant correlations between longitudinal change in cortical parameters (volume, thickness) and longitudinal change in reading.

Fig. 5 shows the significant correlations between the structural parameters and reading: increased volume in the right IFG_orb_ (*r* = .61, p_*fdr*_ =.002), increased cortical thickness in the right IFG_orb_ (*r* = .59, p_*fdr*_ =.002), increased cortical thickness in the left SPC (*r* = .51, *p*_fdr_ =.010) and increased volume in the right ITG (*r* = .48, *p*_fdr_ =.017) all correlated with larger improvements in reading performance.

**Fig. 6:**
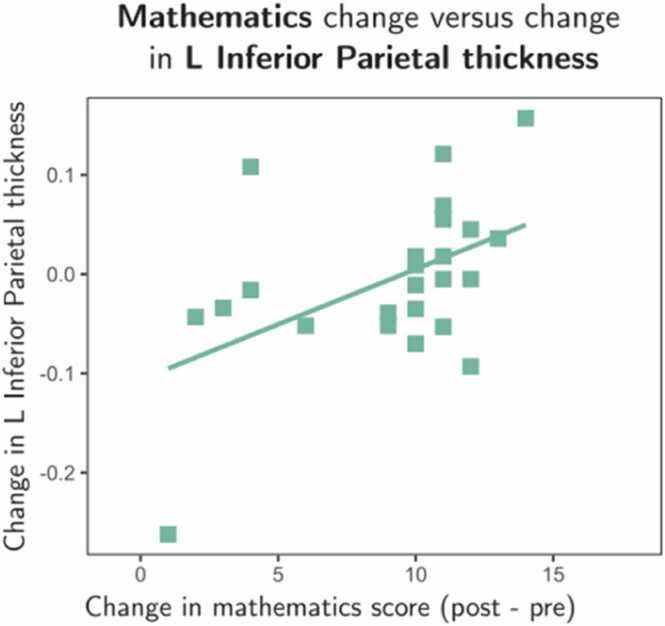
Grouped scatterplot of significant correlation between longitudinal change in left inferior parietal thickness and longitudinal change in mathematics.

For mathematics, we only found one significant correlation: increased **cortical thickness of the left IPC** (*r* = .49, *p*_fdr_ =.016) correlated with larger improvement in mathematical performance (Fig. 6).

## 4 Discussion

This study investigated changes in cortical gray matter in children transitioning from preschool to primary school, aiming to disentangle schooling-induced effects from age-related maturational changes. By leveraging a school cut-off design with two similar-aged groups differing only in formal education exposure, we assessed whether cortical gray matter changes in 5–7-year-old children – particularly in regions linked to reading and mathematics – could be attributed to schooling. Our results showed that one year of schooling resulted in schooling effects in several regions within the bilateral – but mainly left-hemispheric – temporal and parietal cortices and bilateral inferior frontal gyrus. Post-hoc analyses further demonstrated that specific regions within the bilateral temporal, parietal, and inferior frontal gyri were uniquely correlated with behavioral gains in reading and mathematics. These data provide an intriguing example of experience-dependent plasticity and show how the brain structurally changes in response to formal schooling.

Our mixed-effects models revealed that the most pronounced effects of schooling were observed in cortical volume and thickness. This aligns with our hypothesis and prior evidence suggesting that postnatal experiences exert a greater impact on volume and thickness whereas cortical surface area is more strongly influenced by prenatal factors (Jha et al., 2019). The observed schooling effects were localized in bilateral temporal and parietal cortices – predominantly in the left hemisphere – and bilateral inferior frontal gyri (*pars triangularis* and *pars orbitalis*). These regions have been consistently associated with reading and mathematical abilities in primary school children (e.g., Li et al., 2013; Perdue et al., 2020; Suárez-Pellicioni et al., 2021; Xia et al., 2018).

The observed gray matter changes are broadly consistent with the dual-route model of reading (Coltheart et al., 2001, Jobard et al., 2003). This model posits a dorsal phonological decoding pathway linking temporo-parietal and inferior frontal regions, and a ventral orthographic route connecting inferior frontal with occipito-temporal regions (Jobard et al., 2003, Schlaggar and McCandliss, 2007). Although both routes are ultimately expected to exhibit left-hemispheric lateralization, previous research indicates that such lateralization is not yet fully established in young children (Benischek et al., 2020, Raschle et al., 2012, Reynolds et al., 2019, Xiao et al., 2016). Given that our participants were beginning readers, who predominantly rely on letter-by-letter decoding strategies (Frith, 1985), the observed structural changes in temporo-parietal and inferior frontal regions likely reflect an early reliance on the dorsal reading route. However, our findings do not fully align with the traditional dual-route model. First, within the inferior frontal gyrus, the schooling effects were located in the anterior IFG (*pars triangularis* and IFG *pars orbitalis)*, which have typically been associated with lexico-semantic processing along the ventral reading route rather than with phonological processing along the dorsal reading route, where the posterior IFG (*pars opercularis)* is more strongly implicated (Dickens et al., 2019, Heim et al., 2005, Poldrack et al., 1999, Sánchez et al., 2023). Second, we also observed schooling effects on the superior parietal cortex, a region not typically included in the dual-route model (Coltheart et al., 2001, Jobard et al., 2003). Nevertheless, meta-analytic evidence suggests that this region is more engaged during pseudoword compared to word reading, supporting its potential involvement in the dorsal reading route (Taylor et al., 2013). Although the number of comparative longitudinal studies in this developmental window of 5–7-years is limited, our findings align with earlier work on reading which demonstrates that during emergent reading, increases in cortical volume in left-hemispheric temporo-parietal regions are observed (Phan et al., 2021). They are in line with the observation that cortical thickening in the left inferior frontal gyrus correlates with phonological skill development (Lu et al., 2006).

It is important to note that the interaction effects on cortical thickness were primarily driven by cortical thinning in the non-schooling group, whereas no significant change was observed in the schooling group. Thus, the observed interaction reflects an attenuation of normative developmental thinning in the schooling group rather than cortical thickening. Interpreting the cortical thinning observed in the non-schooling group requires careful consideration. First, our data revealed significant main effects of timepoint on cortical thickness across multiple regions, characterized by significant cortical thinning from pre- to posttest. This aligns with prior research indicating that cortical thinning begins as early as age three (Frangou et al., 2022, Sowell, 2004, Walhovd et al., 2016). Although the precise developmental trajectory remains debated (Ducharme et al., 2016, Tamnes et al., 2017, Walhovd et al., 2016), cortical thinning is generally considered a hallmark of general brain maturation. Particularly, developmental thinning is thought to reflect a combination of biological processes, including *synaptic pruning* and increased myelination (Huttenlocher and Dabholkar, 1997, Sakai, 2020). It is hypothesized that underutilized axons are pruned away during childhood, whereas remaining axons undergo increased myelination (Jeon et al., 2015, Natu et al., 2019, Sowell, 2004). Although the precise role of pruning in learning and development remains unclear (Zhou et al., 2015), considerable interindividual variability in the timing and extent of pruning has been proposed as a potential factor underlying differences in cognitive development (Yeatman et al., 2012). Within this developmental context, the absence of thinning in the schooling group may reflect a deceleration of normative thinning trajectories. This interpretation is consistent with prior longitudinal and intervention studies. For example, Kuhl et al. (2020) investigated children before and after they started formal schooling and found that those who showed greater improvements in mathematical performance also exhibited reduced cortical thinning in the right temporal pole and left middle occipital gyrus. Given that both regions typically exhibit age-related cortical thinning during development (Ducharme et al., 2016), this pattern may reflect a deceleration of cortical thinning in children with better mathematical performance, potentially due to increased engagement or training. Similarly, Economou et al. (2024) reported significant cortical thinning in several regions in control groups, whereas children at risk for dyslexia receiving a tablet-based phonics intervention did not show this age-related thinning, suggesting that targeted learning experiences may modulate normative thinning trajectories. Also, in our own correlation analyses conducted within the schooling group, we observed a positive relationship between gains in reading and mathematics ability and higher cortical thickness values in specific cortical regions. This supports the notion that the acquisition of new skills might attenuate normative thinning trajectories (Lövdén et al., 2013). Third, we also observed significant interaction effects on the other cortical parameters, i.e., volume and surface area. These effects were characterized by significant increases in the schooling group and no change in the non-schooling group. Notably, for the left inferior parietal cortex and right inferior frontal gyrus (*pars orbitalis*), schooling effects were found for both volume and thickness – thereby enhancing the robustness of our findings by demonstrating effects across cortical parameters.

Correlational analyses further revealed that changes in reading and mathematics performance were differentially associated with changes in specific cortical regions. Reading gains correlated with increased volume in the right inferior frontal gyrus (*pars orbitalis*) and left superior parietal cortex, and increased thickness in the right inferior frontal gyrus (*pars orbitalis*) and right inferior temporal gyrus. The anterior inferior frontal gyrus (*pars orbitalis* and *pars triangularis*) is typically involved in lexico-semantic processing and controlled language functions during reading (Fiebach et al., 2002, Sánchez et al., 2023). In beginning readers, bilateral inferior frontal engagement has been reported, potentially reflecting increased reliance on semantic information and effortful language processing during initial reading acquisition (Benischek et al., 2020, Reynolds et al., 2019). While the left superior parietal cortex is less frequently discussed in the context of reading, studies have suggested that it may support phonological encoding (Taylor et al., 2013) or alternatively, attentional processes during reading, particularly under conditions of increased task demands (Lobier et al., 2014, Reilhac et al., 2013, Vogel et al., 2013). Supporting this, increased activation in the bilateral superior parietal lobule has been reported in typical readers in response to print (Chyl et al., 2019), and both beginning readers (Wang et al., 2020) and adults (Vogel et al., 2013) show stronger bilateral superior parietal lobule activation for pseudoword as compared to word reading. The inferior temporal gyrus, on the other hand, is typically part of the reading network in beginning readers, showing increased bilateral activity during word reading (Marks et al., 2019). In the right hemisphere, activation levels in response to print (words and pseudowords) have been shown to correlate with behavioral predictors of reading (Pugh et al., 2013). In line with this, we found a significant association between reading gains and increased cortical thickness in the right – but not left – inferior temporal gyrus. This finding aligns with two gray matter intervention studies on reading that also reported training-related effects on cortical thickness in the right inferior temporal gyrus (Economou et al., 2024, Romeo et al., 2018), suggesting that this region may be particularly sensitive to reading-related plasticity in beginning readers. It may seem surprising, however, that no association was found with the left inferior temporal gyrus. For instance, Xia et al. (2018) reported a significant correlation between thickness in the left inferior temporal gyrus and orthography-phonology mapping. Yet, their participants were older (10 – 12 years) and likely more proficient readers. Because the children in the current study were beginning readers, direct comparisons are difficult. It should be noted that the majority of studies on reading investigated older children, in whom the reading network tends to be more left-lateralized and distributed across temporoparietal, inferior frontal, and ventral occipito-temporal regions (Martin et al., 2015, Murphy et al., 2019). Nevertheless, our findings are consistent with the hypothesis that, in beginning readers, the network is more broadly distributed across both hemispheres (Pugh et al., 2013), with initial recruitment and subsequent disengagement of right-hemisphere regions (Shaywitz et al., 2002, Turkeltaub et al., 2003, Yamada et al., 2011).

In mathematics, increases in cortical thickness in the left inferior parietal cortex – including the angular and inferior parietal gyri (Desikan et al., 2006) – were significantly correlated with performance gains. This finding supports longstanding evidence linking the parietal cortex to number processing and quantity representation (Arsalidou et al., 2018, Peters and De Smedt, 2018). Specifically, the left inferior parietal gyrus has been associated with calculation tasks and Arabic digit processing, whereas the right inferior parietal gyrus is thought to facilitate more abstract symbolic number representations and general number processing (Arsalidou et al., 2018, Sokolowski et al., 2017). The angular gyrus, on the other hand, is assumed to contribute to the verbal processing of numbers, particularly in fact retrieval (Dehaene et al., 2003). Recent evidence suggests that its engagement during fact retrieval may stem from a broader computational function or its role in attention (Bloechle et al., 2016, Sokolowski et al., 2023). Considering the developmental literature, which suggests that the angular gyrus becomes increasingly relevant with age due to greater reliance on automatized retrieval strategies (Menon, 2016, Peters and De Smedt, 2018, Polspoel et al., 2017, Rivera et al., 2005), it is plausible that the observed associations primarily reflect contributions from the left inferior parietal gyrus in arithmetic. However, because the Desikan–Killiany atlas (Desikan et al., 2006) combines anatomically adjacent regions within the inferior parietal cortex – the angular and inferior parietal gyri – we cannot directly disentangle their respective contributions. Future studies with more fine-grained parcellations that distinguish between these neighboring gyri in the inferior parietal cortex would provide greater anatomical precision and allow more definitive conclusions regarding their specific roles. Nonetheless, the currently used atlas (Desikan et al., 2006) remains the most widely used in developmental studies of cortical morphometry (e.g., Bethlehem et al., 2022; Collins et al., 2022; Tamnes et al., 2017), and therefore offered the most appropriate framework for ensuring replicability and facilitating comparisons with previous research.

We did not observe schooling-related effects in other regions previously implicated in reading and mathematics, including the fusiform gyrus, supramarginal gyrus and intraparietal sulcus. We briefly consider each of these regions in turn, discussing their known functional roles and the implications of the absence of schooling effects in the context of prior research.

First, no schooling-induced effects were observed in the fusiform gyrus. This may seem surprising because its functional specialization to letter strings is often seen as the anatomical signature of emergent reading (Dehaene and Dehaene-Lambertz, 2016). It is plausible that changes in the functional neural pattern that coincide with learning to read (Dehaene-Lambertz et al., 2018, Dehaene and Cohen, 2011), also imply that brain structures reorganize during this period. However, to the best of our knowledge, only one previous gray matter intervention study on reading found such training-related increases in the left fusiform gyrus (Krafnick et al., 2011), albeit in a small sample (*n* = 11) of older children with reading difficulties (ages 7 – 11). In previous intervention studies with beginning readers, no effects have been found in the left fusiform gyrus (Economou et al., 2024, Romeo et al., 2018).

Second, the (left) supramarginal gyrus is commonly activated during tasks involving phonological processing (Price, 2012), and proposed to be part of the dorsal reading route (Jobard et al., 2003). It has also been shown to be activated during arithmetic (Evans et al., 2016, Peters et al., 2016) and structural differences have been reported between neurotypical children and children with or at risk for learning difficulties, including dyslexia (Economou et al., 2024, Linkersdörfer et al., 2012) and dyscalculia (McCaskey et al., 2020). The current study observed a trend for increased pre- to post-thickness in the left supramarginal gyrus for the schooling group compared to a declining thickness trend in the non-schooling group (*β* = 0.17), but this interaction did not reach conventional levels of statistical significance (*p* = .054).

Lastly, it is important to note that we were unable to specifically investigate schooling effects on the intraparietal sulcus (IPS) in the present study due to limitations of the gyral-based atlas employed (Desikan et al., 2006), which does not delineate the IPS as a distinct ROI, but rather encompasses it under broader parietal regions. The IPS is a well-established region in fMRI studies in mathematics demonstrating consistent specific increases in IPS activity during calculation tasks, suggesting its involvement in numerical magnitude processing (Menon, 2014, Peters and De Smedt, 2018). In addition, longitudinal structural imaging work has shown that gray matter volume in the IPS is associated with gains in arithmetic (Suárez-Pellicioni et al., 2021), further underscoring its relevance for mathematical learning.

Taken together, the number of studies on cortical gray matter at the ages 5-to-7 in neurotypical children is very small. As a result, much remains unclear, and given that changes in brain structure and brain function are not inherently synonymous and can manifest independently from each other (Galván, 2010), it is possible that the functional specialization of the fusiform gyrus for reading or the intraparietal sulcus for mathematics occurs without clear changes in the underlying structural architecture at this time. A similar argument may apply to other regions implicated in mathematical cognition, such as the middle/superior temporal gyri or hippocampus. Much of the evidence linking these regions to arithmetic development stems from functional neuroimaging studies (in older children) (e.g., Cho et al., 2012; Fias et al., 2021; Prado et al., 2014; Qin et al., 2014). In addition, numerical processing relies on a broadly distributed fronto-parietal network rather than being confined to IPS alone (Skagenholt et al., 2018, Sokolowski et al., 2017), further highlighting that functional specialization may occur across multiple regions without necessarily being accompanied by structural changes.

Several limitations must be acknowledged in the current study. Although we observed experience-dependent specific changes in gray matter in response to schooling, the precise neural mechanisms underlying the observed structural changes remain unclear. This is because cortical morphometry is influenced by diverse cellular processes including neuronal size, glial cell density, and vascular changes (Zatorre et al., 2012). Given the very limited evidence on learning-induced gray matter changes in young children, it is difficult to pinpoint which of these processes may have contributed to the observed changes. Future studies should therefore focus on both short- and long-term trajectories of gray matter plasticity across a wide age range to elucidate the underlying neural processes more clearly.

A further limitation concerns the inherent constraints of the school cut-off design. Although this quasi-experimental approach allows relatively strong causal inferences regarding the unique impact of schooling on developmental and academic outcomes (Miller et al., 2016), it does not permit conclusions about which specific components of schooling drive the observed effects (Kim et al., 2021, Vandecruys et al., 2025). Instructional methods, classroom environments, and peer interactions each might play a role, but their relative contributions to the structural brain changes observed remain unclear. Future studies integrating observational data from classroom environments, alongside neuroimaging and behavioral measures, would help to elucidate the mechanisms underlying schooling-related neural plasticity and clarify which aspects of the educational context are most influential. While our design allows us to contrast exposure to primary school versus preschool, it does not allow us to determine whether starting school earlier or later leads to better long-term outcomes. Addressing such questions would require a different educational context in which school-entry timing is systematically varied or manipulated, or long-term follow-up designs that examine later academic trajectories in children who started earlier or later with school.

Another limitation relates to the anatomical specificity and spatial scope of our findings. First, our analyses were restricted to a predefined set of cortical regions selected a priori based on brain imaging literature on reading and mathematics. This hypothesis-driven ROI approach was chosen to directly test theoretically motivated predictions. However, limiting analyses to predefined regions constrains conclusions about anatomical specificity. It also remains possible that formal schooling induces structural changes outside the canonical reading and mathematical networks that were not captured in the present study. Addressing such broader or more distributed effects represents an avenue for future research that is seeking to characterize the broader neuroanatomical impact of formal education.

Further, even within the selected regions, anatomical precision is influenced by the choice of parcellation scheme. Our ROIs were derived from a widely used gyral-based atlas (Desikan et al., 2006) which defines relatively large cortical parcels. Summarizing structural metrics within such parcels as single averaged values means that we might have missed smaller, localized effects. While this atlas has been widely used in previous research on both reading and mathematics (e.g., Collins et al., 2022; Economou et al., 2024; Romeo et al., 2018; Saygin et al., 2016), more fine-grained parcellations are available that provide greater anatomical precision and distinguish between adjacent cortical regions within the inferior parietal cortex, such as the angular and inferior parietal gyri, as well as specific sulci such as the IPS (Destrieux et al., 2010). Interestingly, a recent gray matter intervention study compared both atlases (Desikan et al., 2006, Destrieux et al., 2010) in a sample of 5-to-6-year-olds – the same age range as ours – and concluded that the main findings of the study were independent of the labelling method with only slight variations with respect to precise location and therefore robust to the analysis approach (Economou et al., 2024). This suggests that the current findings would hold if another atlas was used.

Motion artifacts led to the exclusion of 15% of datasets, potentially reducing our statistical power. This is particularly relevant for the correlational analyses that correlated behavioral changes with structural changes. These analyses were restricted to the schooling group, because the non-schooling group had not yet learned to read (Median word reading score = 1 at posttest, see Table 3) and did not receive any formal reading instruction. Despite our best efforts to develop a child-friendly protocol imaging protocol, the rate of excluded scans is in line with previous research with a similar preprocessing protocol (e.g., Blockmans et al., 2023, 15%; Economou et al., 2024, 22%). Undoubtedly, the sample size, dropout and data quality remain major challenges of longitudinal neuroimaging studies in young pediatric samples such as the present one (Turesky et al., 2021).

The present study provides, for the first time, evidence that the transition from preschool to primary school is associated with bilateral changes in cortical gray matter. These changes go beyond maturational trajectories and appear to be linked to emergent reading and mathematics acquired through formal instruction. Future studies employing fine-grained anatomical atlases, larger samples, and multimodal imaging will be crucial to fully understand the neural correlates of early childhood education.

## CRediT authorship contribution statement

**Floor Vandecruys:** Writing – original draft, Validation, Project administration, Methodology, Investigation, Formal analysis, Conceptualization. **Maaike Vandermosten:** Writing – review & editing, Validation, Supervision, Project administration, Methodology, Conceptualization. **Bert De Smedt:** Writing – review & editing, Validation, Supervision, Project administration, Methodology, Funding acquisition, Data curation, Conceptualization.

## Declaration of Generative AI and AI-assisted technologies in the writing process

During the preparation of this work the authors used Microsoft Copilot in order to improve language and readability. After using this tool/service, the authors reviewed and edited the content as needed and take full responsibility for the content of the published article.

## Declaration of Competing Interest

The authors declare the following financial interests/personal relationships which may be considered as potential competing interests: Bert De Smedt reports financial support was provided by Research Foundation Flanders. If there are other authors, they declare that they have no known competing financial interests or personal relationships that could have appeared to influence the work reported in this paper.

## Data Availability

Research data will be made publicly available upon acceptance at the Open Science Framework (OSF) repository of corresponding author Floor Vandecruys.

## Footnotes

Appendix A Supplementary data associated with this article can be found in the online version at doi:10.1016/j.dcn.2026.101749.

## References

[bib1] Andraszewicz S., Scheibehenne B., Rieskamp J., Grasman R., Verhagen J., Wagenmakers E.-J. (2015). An introduction to bayesian hypothesis testing for management research. J. Manag..

[bib2] Arsalidou M., Pawliw-Levac M., Sadeghi M., Pascual-Leone J. (2018). Brain areas associated with numbers and calculations in children: meta-analyses of fMRI studies. Dev. Cogn. Neurosci..

[bib3] Bates D., Mächler M., Bolker B., Walker S. (2015). Fitting Linear Mixed-Effects Models Using lme4. J. Stat. Softw..

[bib4] Beelen C., Vanderauwera J., Wouters J., Vandermosten M., Ghesquière P. (2019). Atypical gray matter in children with dyslexia before the onset of reading instruction. Cortex.

[bib5] Benischek A., Long X., Rohr C.S., Bray S., Dewey D., Lebel C. (2020). Pre-reading language abilities and the brain’s functional reading network in young children. NeuroImage.

[bib6] Benjamini Y., Hochberg Y. (1995). Controlling the false discovery rate: a practical and powerful approach to multiple testing. J. R. Stat. Soc. Ser. B Stat. Methodol..

[bib7] Bernal-Rusiel J.L., Greve D.N., Reuter M., Fischl B., Sabuncu M.R. (2013). Statistical analysis of longitudinal neuroimage data with Linear Mixed Effects models. NeuroImage.

[bib8] Bethlehem R.A.I., Seidlitz J., White S.R., Vogel J.W., Anderson K.M., Adamson C., Adler S., Alexopoulos G.S., Anagnostou E., Areces-Gonzalez A., Astle D.E., Auyeung B., Ayub M., Bae J., Ball G., Baron-Cohen S., Beare R., Bedford S.A., Benegal V., Alexander-Bloch A.F. (2022). Brain charts for the human lifespan. Nature.

[bib9] Blockmans L., Golestani N., Dalboni da Rocha J.L., Wouters J., Ghesquière P., Vandermosten M. (2023). Role of Family Risk and of Pre-Reading Auditory and Neurostructural Measures in Predicting Reading Outcome. Neurobiol. Lang..

[bib10] Bloechle J., Huber S., Bahnmueller J., Rennig J., Willmes K., Cavdaroglu S., Moeller K., Klein E. (2016). Fact learning in complex arithmetic—the role of the angular gyrus revisited. Hum. Brain Mapp..

[bib11] Blumenthal J.D., Zijdenbos A., Molloy E., Giedd J.N. (2002). Motion artifact in magnetic resonance imaging: implications for automated analysis. NeuroImage.

[bib12] Brod G., Bunge S.A., Shing Y.L. (2017). Does One Year of Schooling Improve Children’s Cognitive Control and Alter Associated Brain Activation?. Psychol. Sci..

[bib13] Cho S., Metcalfe A.W.S., Young C.B., Ryali S., Geary D.C., Menon V. (2012). Hippocampal–Prefrontal Engagement and Dynamic Causal Interactions in the Maturation of Children’s Fact Retrieval. J. Cogn. Neurosci..

[bib14] Chyl K., Fraga-González G., Brem S., Jednoróg K. (2021). Brain dynamics of (a)typical reading development—a review of longitudinal studies. Npj Sci. Learn..

[bib15] Chyl K., Kossowski B., Dębska A., Łuniewska M., Marchewka A., Pugh K.R., Jednoróg K. (2019). Reading Acquisition in Children: Developmental Processes and Dyslexia-Specific Effects. J. Am. Acad. Child Adolesc. Psychiatry.

[bib16] Cohen J. (2013).

[bib17] Collins S.E., Thompson D.K., Kelly C.E., Gilchrist C.P., Matthews L.G., Pascoe L., Lee K.J., Inder T.E., Doyle L.W., Cheong J.L.Y., Burnett A.C., Anderson P.J. (2022). Development of regional brain gray matter volume across the first 13 years of life is associated with childhood math computation ability for children born very preterm and full term. Brain Cogn..

[bib18] Coltheart M., Rastle K., Perry C., Langdon R., Ziegler J. (2001). DRC: A dual route cascaded model of visual word recognition and reading aloud. Psychol. Rev..

[bib19] Dale A.M., Fischl B., Sereno M.I. (1999). Cortical Surface-Based Analysis I. Segmentation and surface reconstruction. NeuroImage.

[bib20] Dale A.M., Sereno M.I. (1993). Improved Localizadon of Cortical Activity by Combining EEG and MEG with MRI Cortical Surface Reconstruction: A Linear Approach. J. Cogn. Neurosci..

[bib21] Davidson C., Shing Y.L., McKay C., Rafetseder E., Wijeakumar S. (2023). The first year in formal schooling improves working memory and academic abilities. Dev. Cogn. Neurosci..

[bib22] Davis N., Cannistraci C.J., Rogers B.P., Gatenby J.C., Fuchs L.S., Anderson A.W., Gore J.C. (2009). The neural correlates of calculation ability in children: an fMRI study. Magn. Reson. Imaging.

[bib23] Davis-Kean P.E., Tighe L.A., Waters N.E. (2021). The role of parent educational attainment in parenting and children’s development. Curr. Dir. Psychol. Sci..

[bib24] Dehaene S., Cohen L. (2007). Cultural recycling of cortical maps. Neuron.

[bib25] Dehaene S., Cohen L. (2011). The unique role of the visual word form area in reading. Trends Cogn. Sci..

[bib26] Dehaene S., Dehaene-Lambertz G. (2016). Is the brain prewired for letters?. Nat. Neurosci..

[bib27] Dehaene S., Piazza M., Pinel P., Cohen L. (2003). Three parietal circuits for number processing. Cogn. Neuropsychol..

[bib28] Dehaene-Lambertz G., Monzalvo K., Dehaene S. (2018). The emergence of the visual word form: Longitudinal evolution of category-specific ventral visual areas during reading acquisition. PLOS Biol..

[bib29] Desikan R.S., Ségonne F., Fischl B., Quinn B.T., Dickerson B.C., Blacker D., Buckner R.L., Dale A.M., Maguire R.P., Hyman B.T., Albert M.S., Killiany R.J. (2006). An automated labeling system for subdividing the human cerebral cortex on MRI scans into gyral based regions of interest. NeuroImage.

[bib30] Destrieux C., Fischl B., Dale A., Halgren E. (2010). Automatic parcellation of human cortical gyri and sulci using standard anatomical nomenclature. NeuroImage.

[bib31] Dickens J.V., Fama M.E., DeMarco A.T., Lacey E.H., Friedman R.B., Turkeltaub P.E. (2019). Localization of Phonological and Semantic Contributions to Reading. J. Neurosci..

[bib32] Ducharme S., Albaugh M.D., Nguyen T.-V., Hudziak J.J., Mateos-Pérez J.M., Labbe A., Evans A.C., Karama S. (2016). Trajectories of cortical thickness maturation in normal brain development — The importance of quality control procedures. NeuroImage.

[bib33] Economou M., Vanden Bempt F., Van Herck S., Glatz T., Wouters J., Ghesquière P., Vanderauwera J., Vandermosten M. (2024). Cortical Structure in Pre-Readers at Cognitive Risk for Dyslexia: Baseline Differences and Response to Intervention. Neurobiol. Lang..

[bib34] Evans T.M., Flowers D.L., Luetje M.M., Napoliello E., Eden G.F. (2016). Functional neuroanatomy of arithmetic and word reading and its relationship to age. NeuroImage.

[bib35] Evans T.M., Kochalka J., Ngoon T.J., Wu S.S., Qin S., Battista C., Menon V. (2015). Brain structural integrity and intrinsic functional connectivity forecast 6 year longitudinal growth in children’s numerical abilities. J. Neurosci..

[bib36] Fias W., Sahan M.I., Ansari D., Lyons I.M. (2021). From counting to retrieving: neural networks underlying alphabet arithmetic learning. J. Cogn. Neurosci..

[bib37] Fiebach C.J., Friederici A.D., Müller K., Cramon D.Y. von (2002). fMRI Evidence for Dual Routes to the Mental Lexicon in Visual Word Recognition. J. Cogn. Neurosci..

[bib38] Fischl B. (2012). FreeSurfer. NeuroImage.

[bib39] Fischl B., Dale A.M. (2000). Measuring the thickness of the human cerebral cortex from magnetic resonance images. Proc. Natl. Acad. Sci..

[bib40] Fischl B., Liu A., Dale A.M. (2001). Automated manifold surgery: constructing geometrically accurate and topologically correct models of the human cerebral cortex. IEEE Trans. Med. Imaging.

[bib41] Fischl Bruce, Salat D.H., Busa E., Albert M., Dieterich M., Haselgrove C., van der Kouwe A., Killiany R., Kennedy D., Klaveness S., Montillo A., Makris N., Rosen B., Dale A.M. (2002). Whole Brain Segmentation. Neuron.

[bib42] Fischl Bruce, Sereno M.I., Dale A.M. (1999). Cortical Surface-Based Analysis II: Inflation, Flattening, and a Surface-Based Coordinate System. NeuroImage.

[bib43] Frangou S., Modabbernia A., Williams S.C.R., Papachristou E., Doucet G.E., Agartz I., Aghajani M., Akudjedu T.N., Albajes-Eizagirre A., Alnæs D., Alpert K.I., Andersson M., Andreasen N.C., Andreassen O.A., Asherson P., Banaschewski T., Bargallo N., Baumeister S., Baur-Streubel R., Dima D. (2022). Cortical thickness across the lifespan: Data from 17,075 healthy individuals aged 3–90 years. Hum. Brain Mapp..

[bib44] Frith U. (1985). Beneath the surface of developmental dyslexia. Surf. Dyslexia.

[bib45] Galván A. (2010). Neural plasticity of development and learning. Hum. Brain Mapp..

[bib46] Gennatas E.D., Avants B.B., Wolf D.H., Satterthwaite T.D., Ruparel K., Ciric R., Hakonarson H., Gur R.E., Gur R.C. (2017). Age-related effects and sex differences in gray matter density, volume, mass, and cortical thickness from childhood to young adulthood. J. Neurosci..

[bib47] Haddad E., Pizzagalli F., Zhu A.H., Bhatt R.R., Islam T., Ba Gari I., Dixon D., Thomopoulos S.I., Thompson P.M., Jahanshad N. (2023). Multisite <scp>test–retest</scp> reliability and compatibility of brain metrics derived from <scp>FreeSurfer</scp> versions 7.1, 6.0, and 5. 3. Hum. Brain Mapp..

[bib48] Han X., Jovicich J., Salat D., van der Kouwe A., Quinn B., Czanner S., Busa E., Pacheco J., Albert M., Killiany R., Maguire P., Rosas D., Makris N., Dale A., Dickerson B., Fischl B. (2006). Reliability of MRI-derived measurements of human cerebral cortical thickness: the effects of field strength, scanner upgrade and manufacturer. NeuroImage.

[bib49] Heim S., Alter K., Ischebeck A.K., Amunts K., Eickhoff S.B., Mohlberg H., Zilles K., von Cramon D.Y., Friederici A.D. (2005). The role of the left Brodmann’s areas 44 and 45 in reading words and pseudowords. Cogn. Brain Res..

[bib50] Huttenlocher P.R., Dabholkar A.S. (1997). Regional differences in synaptogenesis in human cerebral cortex. J. Comp. Neurol..

[bib51] Jednoróg K., Altarelli I., Monzalvo K., Fluss J., Dubois J., Billard C., Dehaene-Lambertz G., Ramus F. (2012). The Influence of Socioeconomic Status on Children’s Brain Structure. PLoS ONE.

[bib52] Jednoróg K., Marchewka A., Altarelli I., Monzalvo Lopez A.K., van Ermingen-Marbach M., Grande M., Grabowska A., Heim S., Ramus F. (2015). How reliable are gray matter disruptions in specific reading disability across multiple countries and languages? insights from a large-scale voxel-based morphometry study. Hum. Brain Mapp..

[bib53] Jeon T., Mishra V., Ouyang M., Chen M., Huang H. (2015). Synchronous changes of cortical thickness and corresponding white matter microstructure during brain development accessed by diffusion MRI tractography from parcellated cortex. Front. Neuroanat..

[bib54] Jha S.C., Xia K., Ahn M., Girault J.B., Li G., Wang L., Shen D., Zou F., Zhu H., Styner M., Gilmore J.H., Knickmeyer R.C. (2019). Environmental Influences on Infant Cortical Thickness and Surface Area. Cereb. Cortex.

[bib55] Jobard G., Crivello F., Tzourio-Mazoyer N. (2003). Evaluation of the dual route theory of reading: a metanalysis of 35 neuroimaging studies. NeuroImage.

[bib56] Johnson M.H., de Haan M. (2011). The biology of change. Dev. Cogn. Neurosci..

[bib57] Kapellou O., Counsell S.J., Kennea N., Dyet L., Saeed N., Stark J., Maalouf E., Duggan P., Ajayi-Obe M., Hajnal J., Allsop J.M., Boardman J., Rutherford M.A., Cowan F., Edwards A.D. (2006). Abnormal Cortical Development after Premature Birth Shown by Altered Allometric Scaling of Brain Growth. PLoS Med..

[bib58] Karipidis I.I., Pleisch G., Di Pietro S.V., Fraga-González G., Brem S. (2021). Developmental Trajectories of Letter and Speech Sound Integration During Reading Acquisition. Front. Psychol..

[bib59] Kim M.H., Ahmed S.F., Morrison F.J. (2021). The Effects of Kindergarten and First Grade Schooling on Executive Function and Academic Skill Development: Evidence From a School Cutoff Design. Front. Psychol..

[bib60] Koller M. (2016). robustlmm: An R Package for Robust Estimation of Linear Mixed-Effects Models. J. Stat. Softw..

[bib61] Krafnick A.J., Flowers D.L., Napoliello E.M., Eden G.F. (2011). Gray matter volume changes following reading intervention in dyslexic children. NeuroImage.

[bib62] Kuhl U., Friederici A.D., Skeide M.A., Friederici A.D., Emmrich F., Brauer J., Wilcke A., Neef N., Boltze J., Skeide M., Kirsten H., Schaadt G., Müller B., Kraft I., Czepezauer I., Dörr L. (2020). Early cortical surface plasticity relates to basic mathematical learning. NeuroImage.

[bib63] Kuznetsova A., Brockhoff P.B., Christensen R.H.B. (2017). lmerTest Package: Tests in Linear Mixed Effects Models. J. Stat. Softw..

[bib64] Lebel C., Beaulieu C. (2011). Longitudinal Development of Human Brain Wiring Continues from Childhood into Adulthood. J. Neurosci..

[bib65] Lenth R.V. (2017). emmeans: Estimated Marginal Means, aka Least-Squares Means. CRAN Contrib. Packages.

[bib66] Li Y., Hu Y., Wang Y., Weng J., Chen F. (2013). Individual structural differences in left inferior parietal area are associated with schoolchildrens’ arithmetic scores. Front. Hum. Neurosci..

[bib67] Linkersdörfer J., Jurcoane A., Lindberg S., Kaiser J., Hasselhorn M., Fiebach C.J., Lonnemann J. (2015). The association between gray matter volume and reading proficiency: a longitudinal study of beginning readers. J. Cogn. Neurosci..

[bib68] Linkersdörfer J., Lonnemann J., Lindberg S., Hasselhorn M., Fiebach C.J. (2012). Grey Matter Alterations Co-Localize with Functional Abnormalities in Developmental Dyslexia: An ALE Meta-Analysis. PLoS ONE.

[bib69] Lobier M.A., Peyrin C., Pichat C., Le Bas J.F., Valdois S. (2014). Visual processing of multiple elements in the dyslexic brain: Evidence for a superior parietal dysfunction. Front. Hum. Neurosci..

[bib70] Lövdén M., Wenger E., Mårtensson J., Lindenberger U., Bäckman L. (2013). Structural brain plasticity in adult learning and development. Neurosci. Biobehav. Rev..

[bib71] Lu L., Leonard C., Thompson P., Kan E., Jolley J., Welcome S., Toga A., Sowell E. (2006). Normal Developmental Changes in Inferior Frontal Gray Matter Are Associated with Improvement in Phonological Processing: A Longitudinal MRI Analysis. Cereb. Cortex.

[bib72] Lüdecke D., Ben-Shachar M., Patil I., Makowski D. (2020). Extracting, Computing and Exploring the Parameters of Statistical Models using R. J. Open Source Softw..

[bib73] Marks R.A., Kovelman I., Kepinska O., Oliver M., Xia Z., Haft S.L., Zekelman L., Duong P., Uchikoshi Y., Hancock R., Hoeft F. (2019). Spoken language proficiency predicts print-speech convergence in beginning readers. NeuroImage.

[bib74] Martin A., Schurz M., Kronbichler M., Richlan F. (2015). Reading in the brain of children and adults: a meta-analysis of 40 functional magnetic resonance imaging studies. Hum. Brain Mapp..

[bib75] McCaskey U., von Aster M., O’Gorman R., Kucian K. (2020). Persistent differences in brain structure in developmental dyscalculia: a longitudinal morphometry study. Front. Hum. Neurosci..

[bib76] McKay C., Wijeakumar S., Rafetseder E., Shing Y.L. (2022). Disentangling age and schooling effects on inhibitory control development: an fNIRS investigation. Dev. Sci..

[bib77] Melby-Lervåg M., Lyster S.-A.H., Hulme C. (2012). Phonological skills and their role in learning to read: a meta-analytic review. Psychol. Bull..

[bib78] Menon V. (2014). The Oxford Handbook of Numerical Cognition.

[bib79] Menon V. (2016). Working memory in children’s math learning and its disruption in dyscalculia. Curr. Opin. Behav. Sci..

[bib80] Miller P., Henry D., Votruba-Drzal E. (2016). Strengthening causal inference in developmental research. Child Dev. Perspect..

[bib81] Mills K.L., Goddings A.-L., Herting M.M., Meuwese R., Blakemore S.-J., Crone E.A., Dahl R.E., Güroğlu B., Raznahan A., Sowell E.R., Tamnes C.K. (2016). Structural brain development between childhood and adulthood: convergence across four longitudinal samples. NeuroImage.

[bib82] Morrison F.J., Kim M.H., Connor C.M., Grammer J.K. (2019). The causal impact of schooling on children’s development: lessons for developmental science. Curr. Dir. Psychol. Sci..

[bib83] Mowinckel A.M., Vidal-Piñeiro D. (2020). Visualization of Brain Statistics With R Packages ggseg and ggseg3d. Adv. Methods Pract. Psychol. Sci..

[bib84] Murphy K.A., Jogia J., Talcott J.B. (2019). On the neural basis of word reading: a meta-analysis of fMRI evidence using activation likelihood estimation. J. Neurolinguist..

[bib85] Natu V.S., Gomez J., Barnett M., Jeska B., Kirilina E., Jaeger C., Zhen Z., Cox S., Weiner K.S., Weiskopf N., Grill-Spector K. (2019). Apparent thinning of human visual cortex during childhood is associated with myelination. Proc. Natl. Acad. Sci..

[bib86] Noble K.G., Houston S.M., Brito N.H., Bartsch H., Kan E., Kuperman J.M., Akshoomoff N., Amaral D.G., Bloss C.S., Libiger O., Schork N.J., Murray S.S., Casey B.J., Chang L., Ernst T.M., Frazier J.A., Gruen J.R., Kennedy D.N., Van Zijl P., Sowell E.R. (2015). Family income, parental education and brain structure in children and adolescents. Nat. Neurosci..

[bib87] Nolden S., Brod G., Meyer A.-K., Fandakova Y., Shing Y.L. (2021). Neural Correlates of Successful Memory Encoding in Kindergarten and Early Elementary School Children: Longitudinal Trends and Effects of Schooling. Cereb. Cortex.

[bib88] Panizzon M.S., Fennema-Notestine C., Eyler L.T., Jernigan T.L., Prom-Wormley E., Neale M., Jacobson K., Lyons M.J., Grant M.D., Franz C.E., Xian H., Tsuang M., Fischl B., Seidman L., Dale A., Kremen W.S. (2009). Distinct Genetic Influences on Cortical Surface Area and Cortical Thickness. Cereb. Cortex.

[bib89] Partanen M., Kim D.H.C., Rauscher A., Siegel L.S., Giaschi D.E. (2021). White matter but not grey matter predicts change in reading skills after intervention. Dyslexia.

[bib90] Perdue M.V., Mahaffy K., Vlahcevic K., Wolfman E., Erbeli F., Richlan F., Landi N. (2022). Reading intervention and neuroplasticity: A systematic review and meta-analysis of brain changes associated with reading intervention. Neurosci. & Biobehav. Rev..

[bib91] Perdue M.V., Mednick J., Pugh K.R., Landi N. (2020). Gray Matter Structure Is Associated with Reading Skill in Typically Developing Young Readers. Cereb. Cortex.

[bib92] Peters L., De Smedt B. (2018). Arithmetic in the developing brain: A review of brain imaging studies. Dev. Cogn. Neurosci..

[bib93] Peters L., Polspoel B., Op de Beeck H., De Smedt B. (2016). Brain activity during arithmetic in symbolic and non-symbolic formats in 9-12 year old children. Neuropsychologia.

[bib94] Phan T.Van, Sima D., Smeets D., Ghesquière P., Wouters J., Vandermosten M. (2021). Structural brain dynamics across reading development: A longitudinal <scp>MRI</scp> study from kindergarten to grade 5. Hum. Brain Mapp..

[bib95] Poldrack R.A., Wagner A.D., Prull M.W., Desmond J.E., Glover G.H., Gabrieli J.D.E. (1999). Functional Specialization for Semantic and Phonological Processing in the Left Inferior Prefrontal Cortex. NeuroImage.

[bib96] Polspoel B., Peters L., Vandermosten M., De Smedt B. (2017). Strategy over operation: neural activation in subtraction and multiplication during fact retrieval and procedural strategy use in children. Hum. Brain Mapp..

[bib97] Polspoel B., Vandermosten M., De Smedt B. (2020). The association of grey matter volume and cortical complexity with individual differences in children’s arithmetic fluency. Neuropsychologia.

[bib98] Prado J., Mutreja R., Booth J.R. (2014). Developmental dissociation in the neural responses to simple multiplication and subtraction problems. Dev. Sci..

[bib99] Price C.J. (2012). A review and synthesis of the first 20years of PET and fMRI studies of heard speech, spoken language and reading. NeuroImage.

[bib100] Price G.R., Wilkey E.D., Yeo D.J., Cutting L.E. (2016). The relation between 1st grade grey matter volume and 2nd grade math competence. NeuroImage.

[bib101] Pugh K.R., Landi N., Preston J.L., Mencl W.E., Austin A.C., Sibley D., Fulbright R.K., Seidenberg M.S., Grigorenko E.L., Constable R.T., Molfese P., Frost S.J. (2013). The relationship between phonological and auditory processing and brain organization in beginning readers. Brain Lang..

[bib102] Qin S., Cho S., Chen T., Rosenberg-Lee M., Geary D.C., Menon V. (2014). Hippocampal-neocortical functional reorganization underlies children’s cognitive development. Nat. Neurosci..

[bib103] Qiu D., Tan L.-H., Siok W.-T., Zhou K., Khong P.-L. (2011). Lateralization of the arcuate fasciculus and its differential correlation with reading ability between young learners and experienced readers: A diffusion tensor tractography study in a chinese cohort. Hum. Brain Mapp..

[bib104] R Core Team (2022). https://www.r-project.org/.

[bib105] Raschle N.M., Zuk J., Gaab N. (2012). Functional characteristics of developmental dyslexia in left-hemispheric posterior brain regions predate reading onset. Proc. Natl. Acad. Sci..

[bib106] Reilhac C., Peyrin C., Démonet J.-F., Valdois S. (2013). Role of the superior parietal lobules in letter-identity processing within strings: FMRI evidence from skilled and dyslexicreaders. Neuropsychologia.

[bib107] Reuter M., Rosas H.D., Fischl B. (2010). Highly accurate inverse consistent registration: A robust approach. NeuroImage.

[bib108] Reuter M., Schmansky N.J., Rosas H.D., Fischl B. (2012). Within-subject template estimation for unbiased longitudinal image analysis. NeuroImage.

[bib109] Reynolds J.E., Grohs M.N., Dewey D., Lebel C. (2019). Global and regional white matter development in early childhood. NeuroImage.

[bib110] Reynolds J.E., Long X., Grohs M.N., Dewey D., Lebel C. (2019). Structural and functional asymmetry of the language network emerge in early childhood. Dev. Cogn. Neurosci..

[bib111] Richlan F., Kronbichler M., Wimmer H. (2013). Structural abnormalities in the dyslexic brain: A meta-analysis of voxel-based morphometry studies. Hum. Brain Mapp..

[bib112] Rivera S.M., Reiss A.L., Eckert M.A., Menon V. (2005). Developmental Changes in Mental Arithmetic: Evidence for Increased Functional Specialization in the Left Inferior Parietal Cortex. Cereb. Cortex.

[bib113] Romeo R.R., Christodoulou J.A., Halverson K.K., Murtagh J., Cyr A.B., Schimmel C., Chang P., Hook P.E., Gabrieli J.D.E. (2018). Socioeconomic Status and Reading Disability: Neuroanatomy and Plasticity in Response to Intervention. Cereb. Cortex.

[bib114] Sakai J. (2020). How synaptic pruning shapes neural wiring during development and, possibly, in disease. Proc. Natl. Acad. Sci..

[bib115] Sánchez A., Carreiras M., Paz-Alonso P.M. (2023). Word frequency and reading demands modulate brain activation in the inferior frontal gyrus. Sci. Rep..

[bib116] Saygin Z.M., Osher D.E., Norton E.S., Youssoufian D.A., Beach S.D., Feather J., Gaab N., Gabrieli J.D.E., Kanwisher N. (2016). Connectivity precedes function in the development of the visual word form area. Nat. Neurosci..

[bib117] Schel M.A., Klingberg T. (2017). Specialization of the right intraparietal sulcus for processing mathematics during development. Cereb. Cortex.

[bib118] Schilling K.G., Chad J.A., Chamberland M., Nozais V., Rheault F., Archer D., Li M., Gao Y., Cai L., Del’Acqua F., Newton A., Moyer D., Gore J.C., Lebel C., Landman B.A. (2023). White matter tract microstructure, macrostructure, and associated cortical gray matter morphology across the lifespan. Imaging Neurosci..

[bib119] Schlaggar B.L., McCandliss B.D. (2007). Development of Neural Systems for Reading. Annu. Rev. Neurosci..

[bib120] Segonne F., Pacheco J., Fischl B. (2007). Geometrically Accurate Topology-Correction of Cortical Surfaces Using Nonseparating Loops. IEEE Trans. Med. Imaging.

[bib121] Ségonne F., Dale A.M., Busa E., Glessner M., Salat D., Hahn H.K., Fischl B. (2004). A hybrid approach to the skull stripping problem in MRI. NeuroImage.

[bib122] Semel E., Wiig E., Secord W.A., de Jong J. (2012). Clinical Evaluation of Language Fundamentals Preschool 2 (CELF preschool-2-NL).

[bib123] Shaywitz B.A., Shaywitz S.E., Pugh K.R., Mencl W.E., Fulbright R.K., Skudlarski P., Constable R.T., Marchione K.E., Fletcher J.M., Lyon G.R., Gore J.C. (2002). Disruption of posterior brain systems for reading in children with developmental dyslexia. Biol. Psychiatry.

[bib124] Simon G., Lanoë C., Poirel N., Rossi S., Lubin A., Pineau A., Houdé O. (2013). Dynamics of the Anatomical Changes That Occur in the Brains of Schoolchildren as They Learn to Read. PLoS ONE.

[bib125] Skagenholt M., Träff U., Västfjäll D., Skagerlund K. (2018). Examining the Triple Code Model in numerical cognition: An fMRI study. PLOS ONE.

[bib126] Sled J.G., Zijdenbos A.P., Evans A.C. (1998). A nonparametric method for automatic correction of intensity nonuniformity in MRI data. IEEE Trans. Med. Imaging.

[bib127] Sokolowski H.M., Fias W., Mousa A., Ansari D. (2017). Common and distinct brain regions in both parietal and frontal cortex support symbolic and nonsymbolic number processing in humans: A functional neuroimaging meta-analysis. NeuroImage.

[bib128] Sokolowski H.M., Matejko A.A., Ansari D. (2023). The role of the angular gyrus in arithmetic processing: a literature review. Brain Struct. Funct..

[bib129] Sowell E.R. (2004). Longitudinal Mapping of Cortical Thickness and Brain Growth in Normal Children. J. Neurosci..

[bib130] Steele C.J., Zatorre R.J. (2018). Practice makes plasticity. Nat. Neurosci..

[bib131] Suárez-Pellicioni M., Soylu F., Booth J.R. (2021). Gray matter volume in left intraparietal sulcus predicts longitudinal gains in subtraction skill in elementary school. NeuroImage.

[bib132] Supekar K., Swigart A.G., Tenison C., Jolles D.D., Rosenberg-Lee M., Fuchs L., Menon V. (2013). Neural predictors of individual differences in response to math tutoring in primary-grade school children. Proc. Natl. Acad. Sci..

[bib133] Tamnes C.K., Herting M.M., Goddings A.-L., Meuwese R., Blakemore S.-J., Dahl R.E., Güroğlu B., Raznahan A., Sowell E.R., Crone E.A., Mills K.L. (2017). Development of the Cerebral Cortex across Adolescence: A Multisample Study of Inter-Related Longitudinal Changes in Cortical Volume, Surface Area, and Thickness. J. Neurosci..

[bib134] Tange O. (2018). GNU Parallel.

[bib135] Taylor J.S.H., Rastle K., Davis M.H. (2013). Can cognitive models explain brain activation during word and pseudoword reading? A meta-analysis of 36 neuroimaging studies. Psychol. Bull..

[bib136] Theys C., Wouters J., Ghesquière P. (2014). Diffusion Tensor Imaging and Resting-State Functional MRI-Scanning in 5- and 6-Year-Old Children: Training Protocol and Motion Assessment. PLoS ONE.

[bib137] Torre G.-A.A., Eden G.F. (2019). Relationships between gray matter volume and reading ability in typically developing children, adolescents, and young adults. Dev. Cogn. Neurosci..

[bib138] Turesky T.K., Vanderauwera J., Gaab N. (2021). Imaging the rapidly developing brain: Current challenges for MRI studies in the first five years of life. Dev. Cogn. Neurosci..

[bib139] Turkeltaub P.E., Gareau L., Flowers D.L., Zeffiro T.A., Eden G.F. (2003). Development of neural mechanisms for reading. Nat. Neurosci..

[bib140] Vandecruys F., Vandermosten M., De Smedt B. (2024). The role of formal schooling in the development of children’s reading and arithmetic white matter networks. Dev. Sci..

[bib141] Vandecruys F., Vandermosten M., De Smedt B. (2025). Education as a natural experiment: The effect of schooling on early mathematical and reading abilities and their precursors. J. Educ. Psychol..

[bib142] Vandermosten M., Vanderauwera J., Theys C., De Vos A., Vanvooren S., Sunaert S., Wouters J., Ghesquière P. (2015). A DTI tractography study in pre-readers at risk for dyslexia. Dev. Cogn. Neurosci..

[bib143] Vogel A.C., Petersen S.E., Schlaggar B.L. (2013). Matching is not naming: A direct comparison of lexical manipulations in explicit and implicit reading tasks. Hum. Brain Mapp..

[bib144] Wagner R.K., Torgesen J.K. (1987). The nature of phonological processing and its causal role in the acquisition of reading skills. Psychol. Bull..

[bib145] Walhovd K.B., Fjell A.M., Giedd J., Dale A.M., Brown T.T. (2016). Through Thick and Thin: a Need to Reconcile Contradictory Results on Trajectories in Human Cortical Development. Cereb. Cortex.

[bib146] Walton M., Dewey D., Lebel C. (2018). Brain white matter structure and language ability in preschool-aged children. Brain Lang..

[bib147] Wang F., Karipidis I.I., Pleisch G., Fraga-González G., Brem S. (2020). Development of Print-Speech Integration in the Brain of Beginning Readers With Varying Reading Skills. Front. Hum. Neurosci..

[bib148] Wechsler D. (2011).

[bib149] Wierenga L.M., Langen M., Oranje B., Durston S. (2014). Unique developmental trajectories of cortical thickness and surface area. NeuroImage.

[bib150] Wilkey E.D., Cutting L.E., Price G.R. (2018). Neuroanatomical correlates of performance in a state-wide test of math achievement. Dev. Sci..

[bib151] Willson V.L. (1976). Critical values of the rank-biserial correlation coefficient. Educ. Psychol. Meas..

[bib152] Winkler A.M., Kochunov P., Blangero J., Almasy L., Zilles K., Fox P.T., Duggirala R., Glahn D.C. (2010). Cortical thickness or grey matter volume? The importance of selecting the phenotype for imaging genetics studies. NeuroImage.

[bib153] Xia Z., Zhang L., Hoeft F., Gu B., Gong G., Shu H. (2018). Neural correlates of oral word reading, silent reading comprehension, and cognitive subcomponents. Int. J. Behav. Dev..

[bib154] Xiao Y., Brauer J., Lauckner M., Zhai H., Jia F., Margulies D.S., Friederici A.D. (2016). Development of the Intrinsic Language Network in Preschool Children from Ages 3 to 5 Years. PLOS ONE.

[bib155] Yamada Y., Stevens C., Dow M., Harn B.A., Chard D.J., Neville H.J. (2011). Emergence of the neural network for reading in five-year-old beginning readers of different levels of pre-literacy abilities: An fMRI study. NeuroImage.

[bib156] Yeatman J.D., Dougherty R.F., Ben-Shachar M., Wandell B.A. (2012). Development of white matter and reading skills. Proc. Natl. Acad. Sci..

[bib157] Zatorre R.J., Fields R.D., Johansen-Berg H. (2012). Plasticity in gray and white: neuroimaging changes in brain structure during learning. Nat. Neurosci..

[bib158] Zhou D., Lebel C., Treit S., Evans A., Beaulieu C. (2015). Accelerated longitudinal cortical thinning in adolescence. NeuroImage.

