# Supplementary material for: Experience-dependent cortical plasticity in response to formal schooling: Effects on networks for reading and mathematics
Source: Dev Cogn Neurosci. 2026 May 25;80:101749. doi: 10.1016/j.dcn.2026.101749 (PMC13265700; doi:10.1016/j.dcn.2026.101749)
Supplement: Supplementary file 1 — Supplementary material [file mmc1.docx]

Supplementary materials

Correlations between cortical parameters


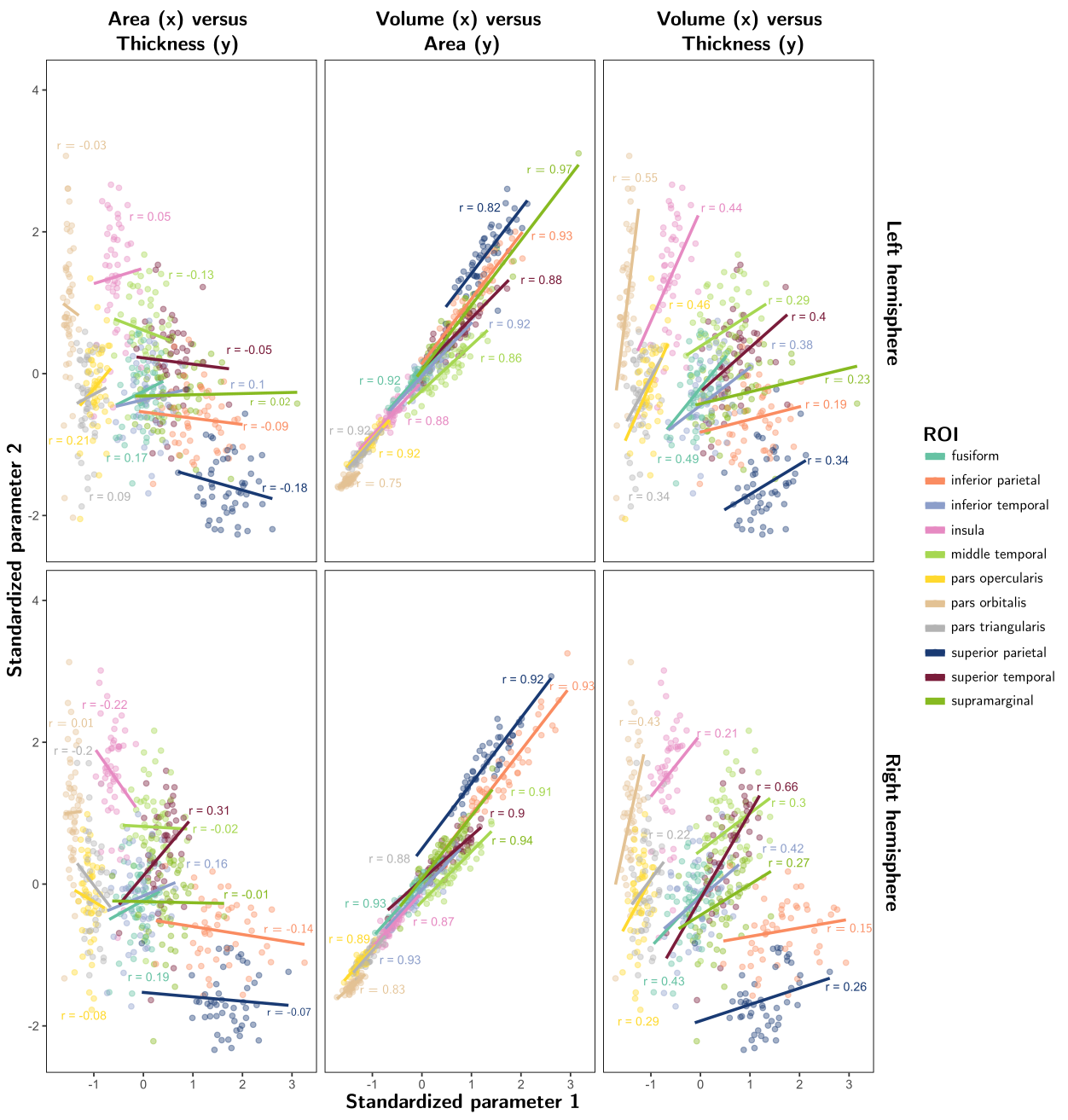


*Supplementary Figure 1 Scatterplots illustrating the correlations between cortical parameters (volume, surface area, thickness). All values were standardized to account for differences in measurement scales and to facilitate comparison. Pearson correlation coefficients are given for each ROI. The hippocampus is not included, as only volume was measured for this region.*

Main effects of timepoint

*Volume*

A main effect of timepoint was found for the **left hippocampus** (*β* = 0.17, 95% CI [0.08, 0.26], *t*(96) = 3.800, *p* < .001), such that both groups had **increased** volume from pre- to posttest.

*Cortical thickness*

Main effects of timepoint were found for the **left IPC** (*β* = -0.30, 95% CI [-0.46, -0.13], *t*(97) = -3.596, *p* < .001), **left IFG_tri_** (*β* = -0.27, 95% CI [-0.42, -0.12], *t*(97) = -3.550, *p* < .001), **left MTG** (*β* = -0.21, 95% CI [-0.35, -0.06], *t*(97) = -2.801, *p* = .005), **right IFG_op_**(*β* = -0.21, 95% CI [-0.38, -0.05], *t*(97) = -2.533, *p* = .011), **right IFG_orb_** (*β* = -0.20, 95% CI [-0.37, -0.03], *t*(97) = -2.400, *p* = .016), **left SMG** (*β* = -0.18, 95% CI [-0.32, -0.04], *t*(97) = -2.515, *p* = .011), **left SPC** (*β* = -0.23, 95% CI [-0.43, -0.04], *t*(97) = -2.364, *p* = .018), **right SPC** (*β* = -0.19, 95% CI [-0.35, -0.03], *t*(97) = -2.295, *p* = .021), **right SMG** (*β* = -0.13, 95% CI [-0.24, -0.01], *t*(97) = -2.174, *p* = .030), **left IFG_op_** (*β* = -0.15, 95% CI [-0.29, -0.01], *t*(97) = -2.018, *p* = .043), **right insula** (*β* = -0.12, 95% CI [-0.25, -0.01], *t*(97) = -2.003, *p* = .045) and **right ITG** (*β* = -0.19, 95% CI [-0.38, -0.01], *t*(97) = -1.991, *p* = .046), indicating that both groups had **decreased** cortical thickness from pre- to posttest.

*Cortical surface area*

Main effects of timepoint were found for the **left insula** (*β* = -0.09, 95% CI [-0.16, -0.02], *t*(96) = -2.533, *p* = .011), **left** **IFG_tri_**(*β* = 0.04, 95% CI [0.01, 0.08], *t*(96) = 2.524, *p* = .011) and **left IFG_op_** (*β* = 0.03, 95% CI [0.01, 0.05], *t*(96) = 2.133, *p* = .032). These main effects indicated a **decrease** from pre- to posttest for the left insula, while an **increase** was demonstrated for the left IFG_tri_ and left IFG_op_.
